# Nitrous oxide produced by denitrifying pseudomonads inhibits the growth of rhizosphere bacteria by inactivating the cobalamin-dependent methionine synthase

**DOI:** 10.1128/mbio.02699-25

**Published:** 2026-03-04

**Authors:** Philip A. Wasson, Darcy L. McRose

**Affiliations:** 1 Department of Civil and Environmental Engineering, Massachusetts Institute of Technology 2167, Cambridge, Massachusetts, USA; 2 Microbiology PhD Program, Massachusetts Institute of Technology 2167, Cambridge, Massachusetts, USA; The University of Oklahoma, Norman, Oklahoma, USA

**Keywords:** microbial communities, nitrous oxide, cobalamin, *Pseudomonas aeruginosa*, denitrification, methionine, rhizosphere, *Arabidopsis thaliana*

## Abstract

Microbial communities are shaped by complex metabolic interactions, whereby the byproducts of one organism influence the physiology of others. This is exemplified in the microbial nitrogen cycle, where diffusion of free intermediates can drastically reshape the chemical landscape of the environment. One such intermediate, nitrous oxide (N_2_O), is often overlooked as biologically inert. However, emerging evidence suggests this gas may inhibit the activity of some cobalamin-dependent enzymes through a reaction with the cofactor. This raises the possibility that, through such an interaction, N_2_O-producing organisms may shape the microbial communities in which they reside, selecting against organisms that rely on these sensitive cobalamin enzymes. At the plant root, a hotspot of microbial activity, the impact of such interactions may be especially important. To investigate this, we focused on microbial N_2_O production and its effect on methionine biosynthesis, a ubiquitous bacterial process carried out by cobalamin-dependent (MetH) or independent (MetE) methyltransferases. In this study, we show that deleting *metE* and forcing reliance on MetH sensitizes the denitrifier *Pseudomonas aeruginosa* to exogenous and self-produced N_2_O. We extend these findings to plant-associated bacteria, where we find that a significant portion of an *Arabidopsis thaliana* rhizosphere culture collection relies exclusively on cobalamin-dependent methionine synthases and experimentally demonstrate their sensitivity to N_2_O. Finally, we show that the growth of one MetH-reliant rhizosphere isolate is suppressed in co-culture with N_2_O-producing *P. aeruginosa*. Together, these findings suggest that N_2_O producers can shape microbial ecology at the plant root.

**IMPORTANCE:**

Microbes that live on plant roots can make important contributions to plant health and often exist in tight-knit communities held together by chemical exchanges. This study investigates an interaction between two such metabolites: the climate-active gas nitrous oxide (N_2_O) and cobalamin. N_2_O can become toxic through a reaction with methionine synthase enzymes that use cobalamin as a cofactor. We asked whether the production of N_2_O by some bacteria curtails the growth of others that rely on these enzymes. Using genetic mutants of a model bacterium and natural isolates from the roots of the plant *Arabidopsis thaliana,* we showed that N_2_O-producing microbes suppress growth of their sensitive neighbors and that N_2_O sensitivity is common in rhizosphere bacteria. As natural and agricultural soils periodically experience bursts of N_2_O, our results suggest that exposure to this gas may shape the assembly of plant-beneficial microbial communities.

## INTRODUCTION

Across environments, microorganisms commonly live in dense consortia where the byproducts of metabolism support and suppress their neighbors. Owing in part to the frequency of diffusible intermediates, the microbial nitrogen cycle has been especially fertile ground for these types of metabolic exchanges (1–4). Nitrous oxide (N_2_O) gas is one such intermediate of particular importance for climate and biogeochemistry (5, 6). Due to its strong greenhouse and ozone-depleting properties, the microbial production and sources of N_2_O have been the subject of intense study (7–13). Despite this, little is known about how elevated concentrations of N_2_O impact microbial communities. This is largely because, unlike nitric oxide (NO), which has been well studied for its widespread cytotoxic effects (14, 15), the more stable N_2_O has typically been considered biologically inert in comparison. However, early on in its use as a dental anesthetic, clinicians studying the safety of the gas became concerned about a side effect: chronic exposure to N_2_O often led to deficiency for cobalamin, a cofactor referred to colloquially as vitamin B_12_ (16, 17). Subsequent biochemical investigations revealed that N_2_O toxicity is potentiated by a reaction with the cobalamin-dependent methionine synthase (MetH), which damages both the enzyme and cofactor (18, 19). While N_2_O toxicity has now been shown in a handful of microorganisms (20–24), its broader ecological consequences remain poorly understood. Considering that amino acid and vitamin exchange and dependencies are common among microbes (25–28), exposure to N_2_O may alter these interactions and represent a previously unappreciated control on microbial community assembly.

**Fig 1:**
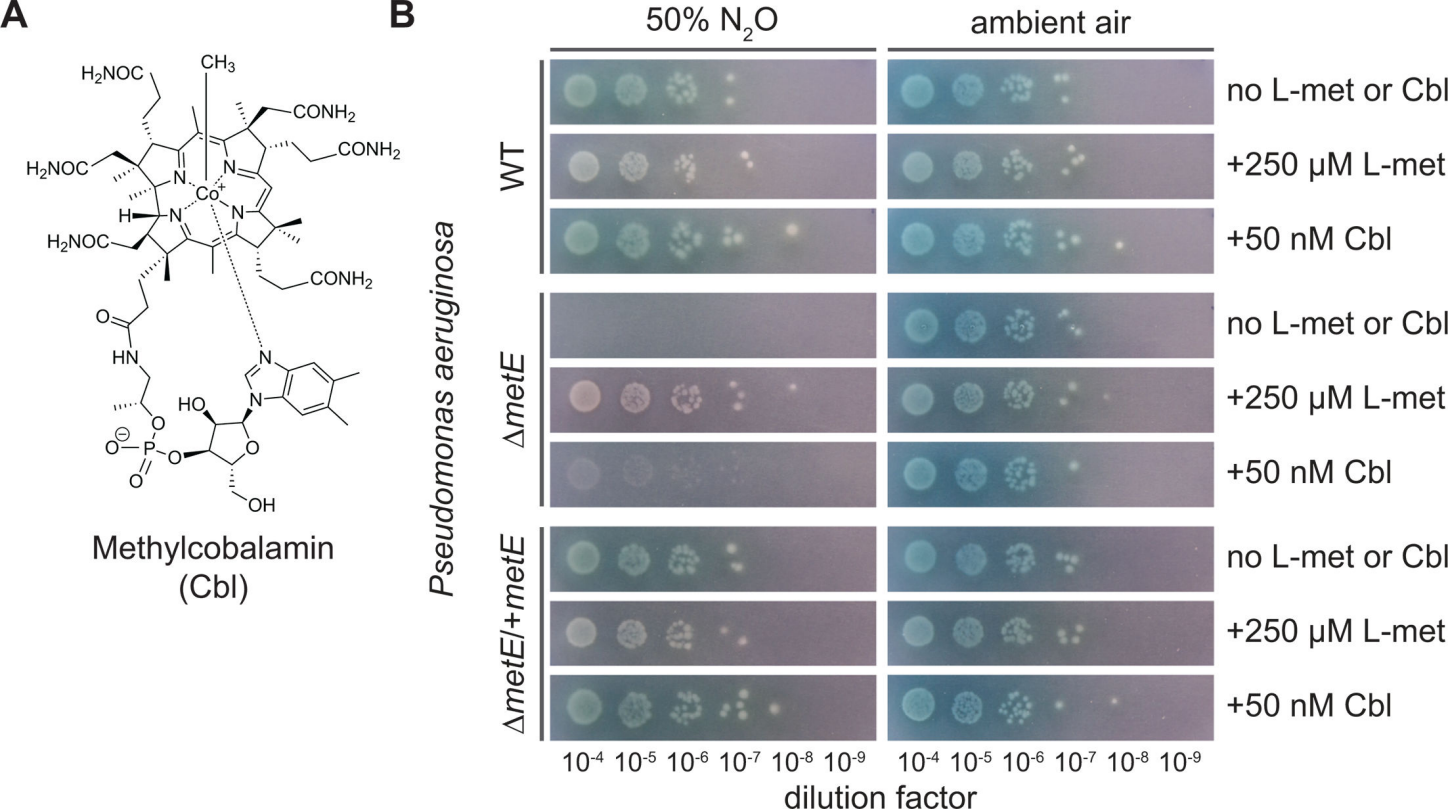
*Pseudomonas aeruginosa* PA14 cobalamin-dependent methionine synthase (MetH) is sensitive to inhibition by N_2_O. (**A**) Structure of cobalamin (Cbl) in its methylated form. The central cobalt atom is in its most oxidized state, Co(III), when coordinated to the methyl group and lower ligand 5,6-dimethylbenzimidazole. (**B**) Growth of *P. aeruginosa* is suppressed compared to wild type (WT) under an atmosphere of approximately 50% N_2_O when the cobalamin-independent methionine synthase is deleted (Δ*metE*), but can be rescued by the addition of 250 µM L-methionine (L-met). Complementation of *metE* (Δ*metE*/+*metE*) also restores growth under high N_2_O.

The link between N_2_O toxicity and cobalamin is thought to occur via the central cobalt (Co) atom in the vitamin (Fig. 1A) (29). Biochemical investigations into MetH have revealed that during the biosynthesis of methionine by MetH, the cofactor’s cobalt atom enters a highly reduced state [Co(I)] that can react with N_2_O (30). It is believed that this reaction generates a damaging oxidant (likely a hydroxyl radical) that modifies both protein and cofactor, inhibiting enzymatic function (19). Interestingly, some bacteria have an alternative methionine synthase (MetE) that does not utilize cobalamin. MetE and MetH show no detectable homology (31, 32), and many organisms encode both forms (33). Recently, an additional group of methionine synthases (MesA, MesB, MesC, and MesD families) has been identified. These four families are believed to be similar to a MetE-ancestor but appear heterogeneous in their reliance on cobalamin, and their sensitivity to N_2_O is unknown (34, 35). Regardless, approximately 30% of sequenced genomes only encode *metH* and hence may be sensitive to the gas (33). Indeed, previous work has shown that deletion of *metE* in *Escherichia coli* (20) and *Paracoccus denitrificans* (21), both of which encode *metE* and *metH*, render these bacteria sensitive to N_2_O, suggesting reliance on MetH (either forced or natural) may be deleterious to bacteria in environments where high concentrations of N_2_O are routinely encountered.

The rhizosphere, or the region of soil directly surrounding plant roots, is one location where this may be consequential. Soils commonly experience elevated N_2_O concentrations (36, 37). This is especially true in agricultural systems, which are the largest anthropogenic sources of N_2_O (5). Here, “hot moments,” or short bursts of very high N_2_O emission, can occur following events that stimulate N_2_O-generating metabolisms (nitrogen fertilization [38], rainfall and thawing [9, 39, 40]) and persist for periods of days to weeks (41–44). Along with nitrification, denitrification is an important source of this N_2_O. When oxygen concentrations are low, denitrifying organisms utilize nitrate (NO_3_^−^) as an alternative electron acceptor, proceeding through a stepwise series of energy-conserving enzymatic reductions (NO_3_^−^ → NO_2_^−^ → NO → N_2_O → N_2_) (45). Complete denitrification terminates in inert dinitrogen gas (N_2_). However, partial denitrification is common and can result in the leakage of N_2_O either because organisms do not encode the terminal nitrous oxide reductase (NosZ) (46–49), or because conditions are not conducive to its activity (inhibition by O_2_ [50], low availability of the metal cofactor: copper [Cu] [51, 52]). Roots are hotspots of denitrification (53–57) and host dense microbial communities that provide essential services to plants, such as pathogen exclusion (58–61) and nutrient acquisition (62–64). We hypothesize that the growth of organisms that only encode the cobalamin-dependent MetH may be abrogated when in close proximity to N_2_O-producing denitrifiers, potentially leading to disruptions in these microbial communities.

To explore this idea, we sought to test whether N_2_O could be inhibitory not just to sensitized producers but also to naturally sensitive neighboring organisms. As a proof of concept, we established co-cultures of N_2_O-sensitive and tolerant microbes using mutant and wild-type strains of *Pseudomonas aeruginosa* UCBPP-PA14 (65). *P. aeruginosa* is a denitrifier that encodes both *metE* and *metH*, allowing this organism to both generate N_2_O and to be sensitized to the gas through deletion of *metE*. We confirmed that *P. aeruginosa* Δ*metE* mutants were sensitive to exogenous, as well as endogenous (self-produced) N_2_O and demonstrated that the co-culture of Δ*metE* mutants with constitutively N_2_O-producing *P. aeruginosa* Δ*nosZ* leads to growth inhibition. To extend these findings to root-associated microorganisms, we leveraged a synthetic rhizosphere community from the model plant *Arabidopsis thaliana* (66). We show that many MetH-reliant rhizosphere isolates are sensitive to N_2_O and demonstrate that the growth of one isolate decreases in co-culture with N_2_O-producing *P. aeruginosa*. Together, our results indicate that N_2_O can shape the composition of microbial communities, including those living at the roots of plants.

## RESULTS AND DISCUSSION

### Deletion of the cobalamin-independent *metE* sensitizes *Pseudomonas aeruginosa* to exogenous N_2_O

To test whether *P. aeruginosa* can be sensitized to N_2_O via inactivation of the cobalamin-dependent methionine synthase, we constructed a Δ*metE* strain and examined its growth on agar plates under an atmosphere of approximately 50% N_2_O. Consistent with previous reports of N_2_O sensitivity in *metE* deficient*-E. coli* and *P. denitrificans*, colony formation by the *P. aeruginosa* Δ*metE* strain was completely inhibited in the presence of N_2_O but not under ambient air (Fig. 1B; Fig. S1A). In contrast, wild-type (WT) *P. aeruginosa* showed no growth defect under a high N_2_O atmosphere. Complementation of *metE* in the mutant strain at the *att*Tn7 site fully restored the ability to grow under a high N_2_O atmosphere (Fig. 1B). Supplementation with 250 µM L-methionine also restored growth of Δ*metE* to WT levels under N_2_O, indicating that methionine biosynthesis is the primary essential process inhibited by N_2_O exposure. Notably, the addition of 50 nM cobalamin did not fully rescue mutant growth, possibly due to the insufficient rate of uptake and holoenzyme assembly relative to the rate of N_2_O-mediated inhibition at these high concentrations of N_2_O. Our method of introducing N_2_O halved the concentration of O_2_ from atmospheric to approximately 11%. To ensure that the growth phenotype we observed was due to the presence of N_2_O and not a decrease in O_2_, we repeated this experiment, using the inert gas N_2_ instead of N_2_O. These experiments showed no growth inhibition for either the mutant or WT strain (Fig. S1B). Together, these results suggest that *P. aeruginosa* can be sensitized to exogenous N_2_O through forced use of the MetH enzyme.

### Copper and nitrate availability influence the accumulation of N_2_O in denitrifying *P. aeruginosa* cultures

**Fig 2:**
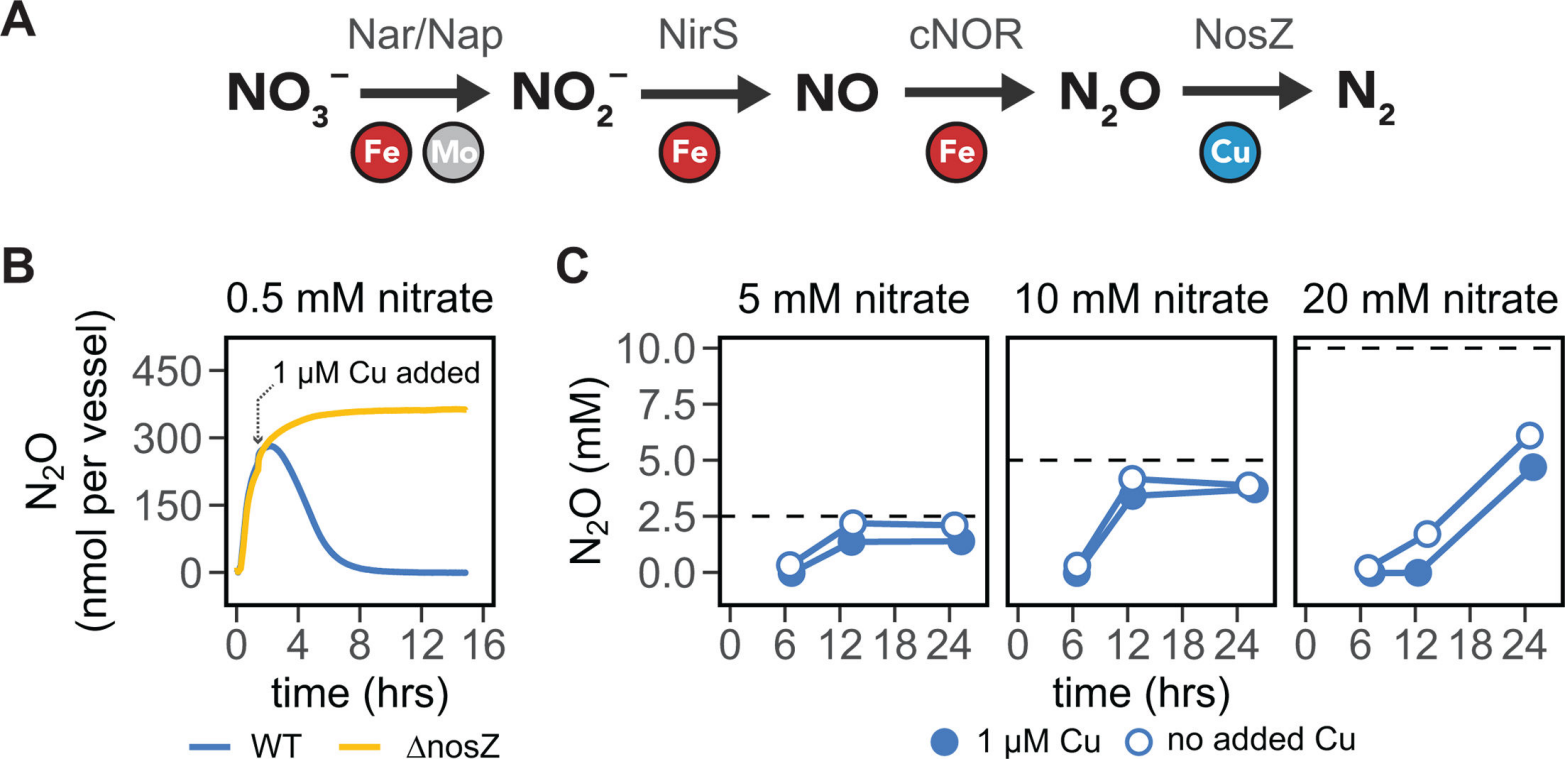
Nitrate and copper availability affect N_2_O accumulation in denitrifying *P. aeruginosa* cultures. (**A**) Schematic of the denitrification pathway in *P. aeruginosa*. Metal cofactor requirements for each reductase are depicted for each step. (**B**) The addition of 1 µM Cu to a Cu-limited *P. aeruginosa* WT cell suspension (OD500 = 1.0) induces the complete consumption of N_2_O from the headspace. *P. aeruginosa* Δ*nosZ* is unaffected by the addition of Cu. N_2_O was measured in the headspace and adjusted using the Henry’s law constant (see Supplemental methods). (**C**) Nitrate concentration in the media of growing *P. aeruginosa* cultures sets the amount of N_2_O accumulated over 24 h. During growth, the addition of Cu delays but does not prevent the accumulation of N_2_O. Points represent the mean of two biological replicates; a replicate experiment can be found in Fig. S3. Error bars, which may be obscured by the data point, depict ±SD. Horizontal dashed lines indicate the theoretical maximum yield of N_2_O that can be generated from a given NO_3_^−^ concentration. N_2_O values reported come from headspace measurements (**B**) or direct measurements of dissolved N_2_O in the aqueous phase (**C**). See Fig. S2 for additional experiments with Cu concentrations from 10 nM to 1 µM.

We were next interested in whether endogenous (self-produced) N_2_O could also inhibit the growth of Δ*metE* mutants. To test this, we first sought to identify conditions that promote N_2_O production and accumulation by *P. aeruginosa*. Here, we took advantage of the fact that the terminal N_2_O reductase (NosZ) is the only enzyme in the *P. aeruginosa* denitrification pathway that requires Cu as a cofactor (Fig. 2A) (45). We reasoned that Cu availability might serve as one way to tune N_2_O accumulation from *P. aeruginosa* (51, 67, 68). Under denitrifying conditions (anoxic with 0.5 mM NO_3_^−^ as the electron acceptor) in the absence of added Cu, WT *P. aeruginosa* rapidly emitted N_2_O at a rate similar to the Δ*nosZ* mutant. The addition of Cu led to N_2_O consumption in WT but not Δ*nosZ* strains (Fig. 2B; Fig. S2). Together, this indicates that the activity of NosZ, and hence N_2_O accumulation, could be manipulated by the availability of Cu.

The availability of NO_3_^−^, a substrate for denitrification, may also influence N_2_O accumulation in denitrifying cultures. To explore this, we measured the accumulation of dissolved N_2_O in anoxic cultures grown with 5, 10, or 20 mM NO_3_^−^ serving as the electron acceptor and 37 mM succinate as the electron donor. Cultures supplied with 5 or 10 mM NO_3_^−^ produced less N_2_O than those supplied with 20 mM NO_3_^−^ and appeared to exhaust NO_3_^−^ by 12 h, showing near full conversion to N_2_O in the absence of copper (Fig. 2C). The addition of 1 µM Cu delayed, but did not prevent, the onset of N_2_O emission. Replicate experiments and experiments in non-sealed growth vessels showed similar differences in N_2_O accumulation (Fig. S3 and S4). This phenotype could be driven by the exhaustion of Cu over these longer experiments. However, considering we did not observe Cu limitation of growth in WT cells, it is more plausible that Cu has a complex regulatory role in controlling NosZ function. One possible explanation for the observed effect of NO_3_^−^ on N_2_O is that the NO reductase outpaces the N_2_O reductase, allowing for leakage of N_2_O during denitrification. However, short-term incubation experiments showed that per cell N_2_O production was similar across NO_3_^−^ concentrations (Fig. S4A), suggesting instead that N_2_O accumulation is set by the N available for conversion to N_2_O. It is also notable that the expression patterns of denitrification enzymes are thought to occur sequentially rather than in tandem (1, 4), which could explain the apparent lack of N_2_O consumption observed even in the presence of Cu (Fig. 2C). Nonetheless, these experiments suggest that there are marked differences between N_2_O accumulation across NO_3_^−^ concentrations, and to a lesser extent Cu levels, and that this can be used to tune gas production.

### *P. aeruginosa* Δ*metE* growth is inhibited under denitrifying conditions that promote N_2_O accumulation

**Fig 3:**
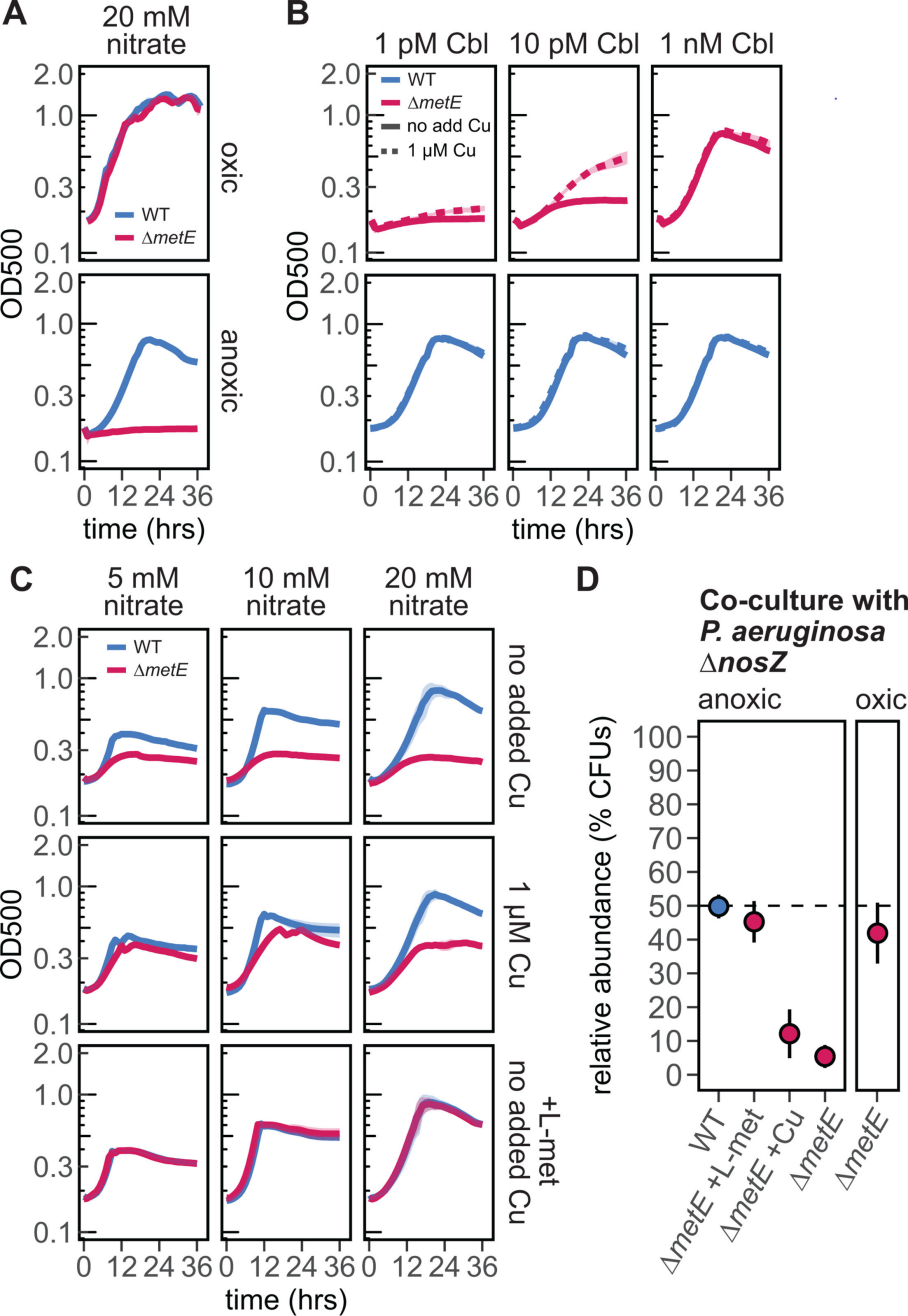
The growth of *P. aeruginosa* Δ*metE* is inhibited by physiological levels of N_2_O. N_2_O produced during denitrification limits the growth of Δ*metE* alone (**A to C**) and in co-culture (**D**). (**A**) A growth defect in Δ*metE* is observed under anoxic but not oxic conditions (with high NO_3_^−^ and low Cu). (**B**) The growth defect of Δ*metE* during anoxic growth can be rescued by the addition of cobalamin (Cbl), but the extent of rescue depends on N_2_O levels as controlled by Cu concentration. (**C**) The Δ*metE* growth defect (with respect to WT) is maximized under conditions that promote high N_2_O accumulation (high NO_3_^−^ and low Cu) and nearly eliminated under conditions with lower N_2_O (low NO_3_^−^ and high Cu). The addition of 250 µM L-methionine (L-met) restores Δ*metE* growth to WT levels regardless of condition. (**D**) In co-culture with *P. aeruginosa* Δ*nosZ*, the fitness of Δ*metE*, as determined by colony-forming units (CFUs), decreased under anoxic conditions (as compared to the same pairing in oxic conditions). No decrease was observed for *P. aeruginosa* WT in anoxic co-culture with Δ*nosZ*. The addition of 250 µM L-methionine rescues Δ*metE*, while Cu has a marginal effect. All cultures in panels **C** and **D** were supplemented with 10 pM cobalamin. Lines in panels **A**, **B**, and **C** represent the mean of three replicates, and points in panel **D** represent the mean of five replicates, with shaded areas and error bars indicating ±SD (may not be visible).

To establish that endogenous N_2_O can be inhibitory to Δ*metE* mutants, we compared the growth of mutant and WT *P. aeruginosa* under conditions shown previously to promote production of the gas. In anoxic conditions with no added Cu and high NO_3_^−^ (20 mM, see Fig. 2C), we observed severe growth inhibition of Δ*metE* (Fig. 3A). In comparison, Δ*metE* showed no growth defect relative to WT under oxic conditions, where denitrification is not expected to occur. These results suggest that a reaction between endogenously produced N_2_O and cobalamin is responsible for the observed phenotypes. However, there is some complexity, as changes in the capacity to produce cobalamin *de novo* under these two conditions could also explain these results. Indeed, there is evidence of an aerobic/anaerobic branching of the pathways used to assemble the corrin ring component of cobalamin (69). Genomic surveys suggest that *P. aeruginosa* encodes many genes predicted to be in both pathways. However, previous authors make note that these annotations remain a poor predictor of these pathways’ overall function (33), and experimental evidence suggests that *P. aeruginosa* may not be able to carry out anaerobic biosynthesis of the vitamin (70, 71). To circumvent potential growth defects due solely to inadequate cobalamin biosynthesis, we designed an experiment in which cobalamin was supplemented at varying concentrations and asked whether endogenously produced N_2_O (as tuned by Cu supply) impacted the benefits of the added cofactor. Under replete Cu, progressive additions of cobalamin led to concomitant increases in mutant growth relative to WT, with full rescue of growth when 1 nM cobalamin was added (Fig. 3B; Fig. S5). However, the relative benefits of these cobalamin additions were tempered under low Cu conditions, where we expect higher N_2_O. In contrast, WT cultures showed no response to cobalamin or Cu. These results suggest that the observed phenotypes depend on N_2_O and cannot be explained solely by differences in cobalamin biosynthetic capacity. All subsequent *P. aeruginosa* growth experiments (Fig. 3C and D) were conducted with 10 pM cobalamin, a concentration at which the effect of N_2_O can be observed phenotypically (Fig. 3B; Fig. S5).

As a second test, we took advantage of the expected variation in N_2_O production during growth on different amounts of NO_3_^−^ (as seen in Fig. 2C). Here, we added progressively higher amounts of NO_3_^−^ in the presence or absence of Cu, with the expectation that in this case, the relative gap between WT and *ΔmetE* might become larger as NO_3_^−^ and presumably N_2_O increased. As expected, under low nitrate (5 mM) and replete Cu (1 µM), the difference between WT and Δ*metE* yield was minimal (Fig. 3C). As NO_3_^−^ concentrations increased, WT but not Δ*metE* yields increased with the most pronounced difference being under low Cu. The addition of 250 µM L-methionine completely restored Δ*metE* growth to WT levels under all conditions. Overall, these results suggest that MetH activity can be inhibited under physiological concentrations of N_2_O produced endogenously during denitrification.

### Reduced fitness of *P. aeruginosa* Δ*metE* in N_2_O-accumulating co-cultures

We hypothesize that N_2_O production by denitrifying organisms may prevent the growth of co-occurring microbes that are sensitive to the gas. As a proof of concept, we paired *P. aeruginosa* Δ*nosZ*, which constitutively produces N_2_O, with either *P. aeruginosa* WT (N_2_O-insensitive) or *P. aeruginosa* Δ*metE* (N_2_O-sensitive) at a starting ratio of 1:1. To monitor changes in relative abundance, we introduced a gentamicin marker into the WT and Δ*metE* strains and quantified growth using selective plating and colony-forming unit (CFU) counting.

Under oxic conditions, the relative abundance of *P. aeruginosa* Δ*metE* after 24 h of co-culture growth with *P. aeruginosa* Δ*nosZ* did not depart significantly from the inoculum ratio of 50% (mean relative abundance of 41.9% ± 8.98%; two-tailed one-sample *t*-test: *t* = −2.02, df = 4, *P* = 0.114) indicating that the deletion of *metE* imparted no fitness defect under conditions where we expect N_2_O accumulation to be low (Fig. 3D). Additionally, this demonstrates that the gentamicin resistance marker used to track Δ*metE* had no unintended effect on fitness. In contrast, under anoxic conditions conducive to denitrification and N_2_O accumulation (20 mM NO_3_^−^ as the electron acceptor), *P. aeruginosa* Δ*metE* exhibited a severe fitness defect in co-culture with *P. aeruginosa* Δ*nosZ*, with a mean relative abundance of just 5.40% ± 3.40%. This defect was fully restored through supplementation with L-methionine. When grown in co-culture with *P. aeruginosa* Δ*nosZ* under the same anoxic conditions, *P. aeruginosa* WT showed no significant departure from the initial inoculum (49.8% ± 3.51%). Because *P. aeruginosa* Δ*metE* may also produce some N_2_O, its decreased fitness could be the sum of endogenous (Δ*metE* produced) and exogenous (Δ*nosZ* produced) N_2_O in the co-culture. The addition of Cu, which should decrease N_2_O production by Δ*metE* but not Δ*nosZ*, led to a small but not significant increase in Δ*metE* abundance, implying that self-produced N_2_O may contribute to the observed phenotypes but does not fully explain them. These data suggest that when nitrate is supplied at high levels, N_2_O produced by denitrifying bacteria can reach high enough concentrations to have deleterious effects on sensitive co-occurring organisms.

### Reliance on MetH is prevalent among *Arabidopsis thaliana* rhizosphere microbiome isolates and largely predicts N_2_O sensitivity

**Fig 4:**
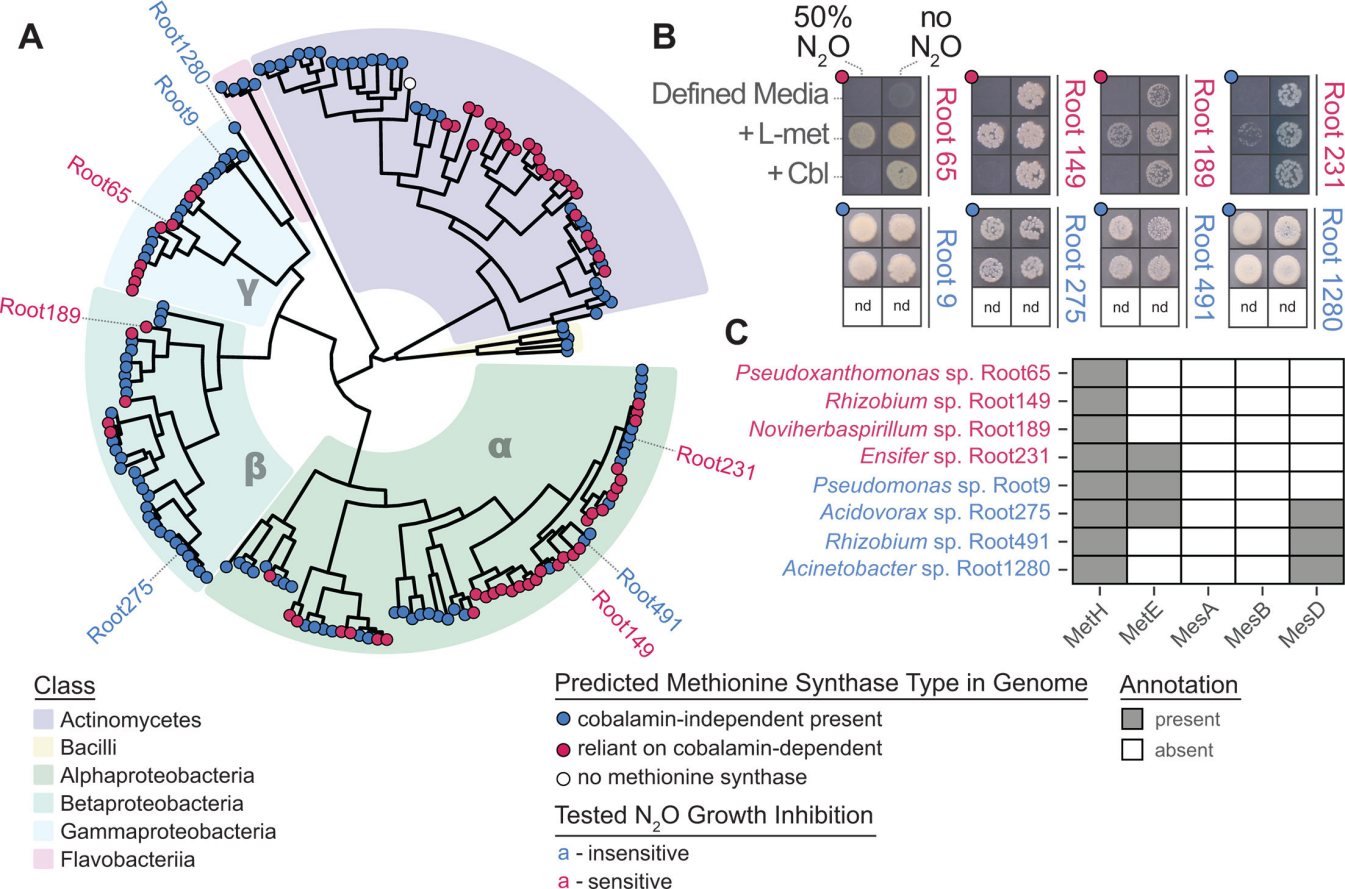
*Arabidopsis thaliana* rhizosphere bacterial isolates are sensitive to N_2_O. (**A**) Phylogenomic tree of the 188-member At-RSPHERE culture collection built from an alignment of a 74 single-copy bacterial gene set (see Materials and Methods). Tip points denote whether the organism relies on cobalamin-dependent (red), cobalamin-independent (blue), or lacks methionine synthases (white) based on predicted genomic annotations. Labeled tips indicate bacteria tested for growth inhibition under an atmosphere of elevated N_2_O with color depicting sensitivity (red) or tolerance (blue). (**B**) Composite images of bacterial colonies after growth under 50% N_2_O or ambient air show the inhibition of Root65, Root149, and Root189, consistent with their reliance on MetH. Strains that did not exhibit a growth defect under a high N_2_O atmosphere were not tested on plates supplemented with cobalamin (nd = no data). (**C**) The genomic annotations of the tested At-RSPHERE isolates are largely consistent with predicted sensitivity (see Fig. S6A through C and Table S3).

All demonstrations of MetH N_2_O sensitivity to date have relied on deletion of *metE* to force reliance on MetH. However, broad genomic surveys indicate that approximately 30% of bacteria encode *metH* as their only methionine synthase (33) and may be sensitive to N_2_O. To explore whether this might happen in the rhizosphere, we took advantage of an established culture collection of bacterial isolates from the roots of the model plant *Arabidopsis thaliana* (66). We searched the genomes of these isolates for methyltransferases involved in methionine biosynthesis, distinguishing between cobalamin-dependent (*metH*, *mesA*, *mesB*, *mesC*) and independent (*metE*, *mesD*) annotations. With one exception (*Yonghaparkia* sp. Root332), all bacteria were predicted to encode at least one methionine synthase (see Table S3). All Flavobacteriia and Bacilli in the collection encode at least one cobalamin-independent methionine synthase. However, consistent with broader surveys, 36% of genomes (68/188) encode only a cobalamin-dependent form. Some members of the α-, β-, and γ-Proteobacteria, as well as Actinomycetes, encode only *metH* and *mesB* (Fig. 4A) and certain genera were populated entirely by taxa that rely solely on cobalamin-dependent methionine synthases (Fig. S6A).

To experimentally verify these predictions, eight strains from across the α-, β-, and γ-Proteobacteria, with five predicted to be tolerant and three predicted to be sensitive, were assayed for growth under approximately 50% N_2_O (Fig. 4A through C). Among the five putatively tolerant strains, four matched their genomic predictions. Interestingly, *Acinetobacter* sp. Root1280 and *Rhizobium* sp. Root491 appear to escape N_2_O toxicity using homologs of the methionine synthase MesD. One putatively tolerant strain showed sensitivity: *Ensifer* sp. Root231. This unexpected sensitivity may reflect a non-functional *metE* protein, low *metE* expression, or possibly the inhibition of other essential Co(I)-dependent cellular processes (72).

Genomic predictions for N_2_O sensitivity showed complete fidelity to experimental results. *Rhizobium* sp. Root149, *Noviherbaspirillum* sp. Root189, and *Pseudoxanthomonas* sp. Root65 were predicted to rely solely on MetH and exhibited severe growth defects under elevated N_2_O that were rescued by the addition of L-methionine (Fig. 4B). At this high level of N_2_O, the addition of cobalamin did not rescue the growth of any sensitive isolate. Notably, *Pseudoxanthomonas* sp. Root65 only grew (regardless of atmosphere) when supplemented with cobalamin or L-methionine and was subsequently confirmed to be a cobalamin auxotroph (EC50 = 87.7 pM; Fig. S7). Together, these data demonstrate that the rhizosphere hosts bacteria that are sensitive to N_2_O-induced growth inhibition and that this can largely be predicted from genomic sequences. Further experimentation focused on understanding the response of *Pseudoxanthomonas* sp. Root65 (which seems to be especially vulnerable as this isolate cannot resynthesize cobalamin damaged in the reaction with N_2_O [19]) to co-culture with an N_2_O producer.

### The fitness of the rhizosphere isolate *Pseudoxanthomonas* sp. Root65 decreases when in co-culture with N_2_O-producing *P. aeruginosa*

As a final test of potential interactions between denitrifiers and resident rhizosphere bacteria, we used qPCR and CFU counts to investigate whether the growth of the naturally sensitive isolate *Pseudoxanthomonas* sp. Root65 could be inhibited by *P. aeruginosa*-produced N_2_O. As *Pseudoxanthomonas* sp. Root65 is an obligate aerobe, co-cultures were conducted under atmospheric oxygen without shaking, thus allowing for the establishment of hypoxic conditions that could simultaneously provide oxygen levels high enough to enable growth of *Pseudoxanthomonas* sp. Root65 but low enough to stimulate denitrification by *P. aeruginosa*. Preliminary experiments confirmed that this experimental setup led to progressive increases in N_2_O in response to different levels of NO_3_^−^ (Fig. S8).

**Fig 5:**
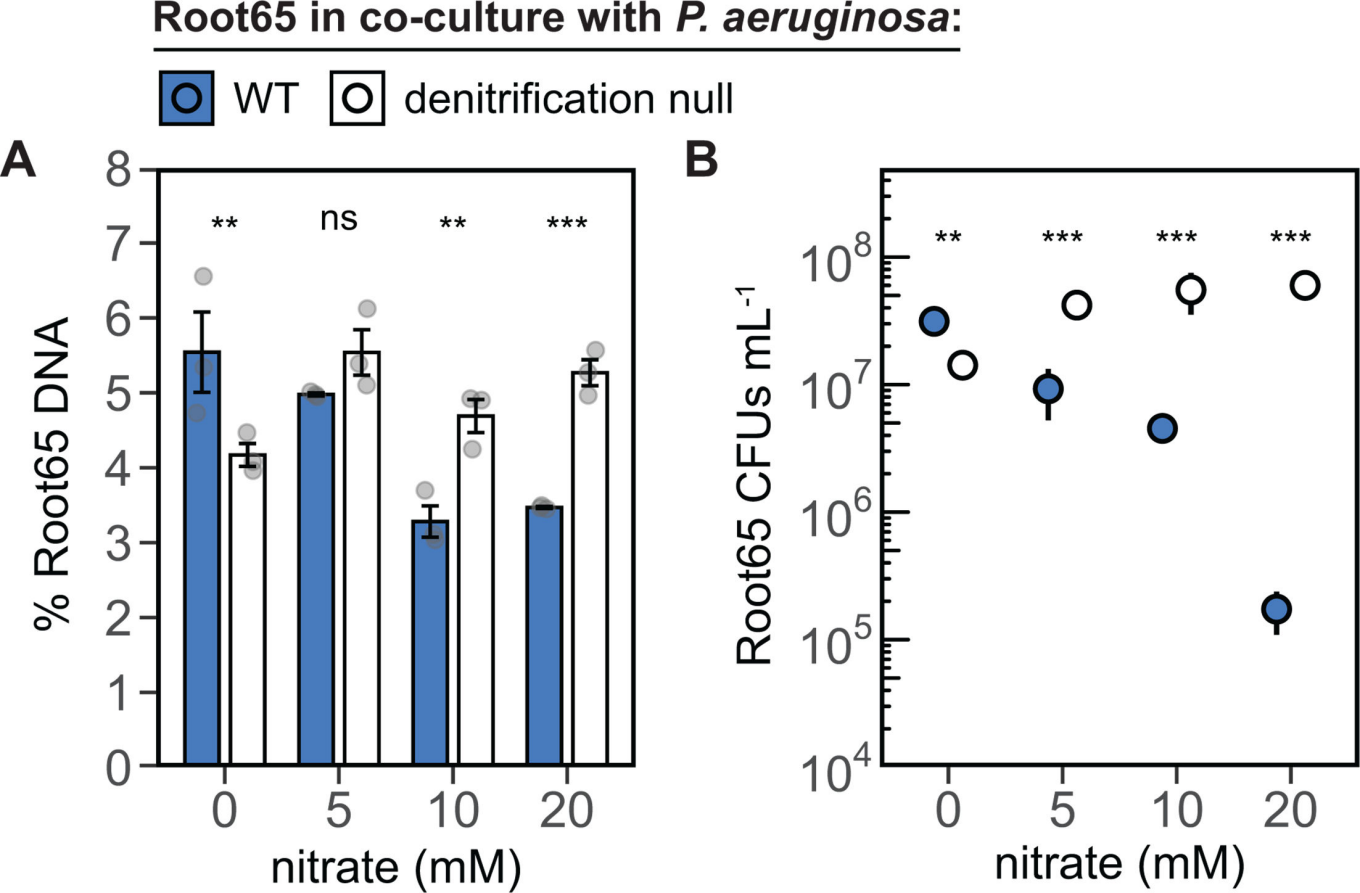
*Pseudoxanthomonas* sp. Root65 abundance decreases in co-culture with N_2_O-producing *P. aeruginosa*. *Pseudoxanthomonas* sp. Root65 abundance was investigated after 24 h of growth in co-culture with *P. aeruginosa* WT (blue) or a denitrification null *P. aeruginosa* mutant (white). The abundance of *Pseudoxanthomonas* sp. Root65 was estimated by qPCR of a species-specific single-copy gene, *secY* (**A**) or by quantification of viable CFUs on selective agar plates (**B**). Bars (**A**) and points (**B**) represent the mean of three biological replicates. Error bars show the standard error of the mean (SEM) (**A**) or the standard deviation (**B**). Statistical analyses were performed using a two-way ANOVA with factors nitrate and paired *P. aeruginosa* strain, followed by Tukey-adjusted pairwise comparisons at each nitrate level. Significant differences are indicated with asterisks (*P < 0.05; **P < 0.01; ***P < 0.001).

After 24 h of co-culture with *P. aeruginosa* under these conditions, we observed a clear inverse relationship between *Pseudoxanthomonas* sp. Root65 abundance and nitrate concentration. Using qPCR primers targeting the single copy gene *secY* in both strains, the percent of *Pseudoxanthomonas* sp. Root65 DNA recovered relative to *P. aeruginosa* was found to decrease by 37.4% from 0 to 20 mM NO_3_^−^ (5.54% ± 0.536% to 3.47% ± 0.013%) (Fig. 5A). As DNA presence does not reflect cell viability, we also conducted CFU counts which showed a much starker decrease of more than two orders of magnitude, from 3.13 × 10^7^ to 1.73 × 10^5^ CFU mL^−1^, across the nitrate gradient (Fig. 5B). Importantly, we did not observe this same decrease when *Pseudoxanthomonas* sp. Root65 was co-cultured with a denitrification null mutant incapable of producing N_2_O (*P. aeruginosa* Δ*narGHJI*, Δ*nirS*, Δ*norBC*, Δ*nosZ*, Δ*fhp* [73]) demonstrating that this phenotype cannot be attributed to other *P. aeruginosa* processes, such as the production of antibiotics or virulence factors, that might correlate with increasing NO_3_^−^. Additionally, *Pseudoxanthomonas* sp. Root65 reached similar yields across all NO_3_^−^ concentrations in mono-culture (Fig. S9E), indicating that nitrogen preference could not explain the differences in growth observed in co-culture. Yields for *P. aeruginosa* WT and denitrification null strains were also stable across nitrate concentrations in both mono- and co-cultures (Fig. S9). Together, these findings suggest that the production of N_2_O by denitrifiers can inhibit the growth of sensitive co-occurring rhizosphere bacteria.

### Implications for rhizosphere microbiome assembly and stability under high N_2_O

Nitrous oxide is a microbially generated gas long studied for its effects on climate but only more recently considered as a player in microbial interactions. Building on long-standing findings from the medical literature (17, 74–76), we show that the production of this gas by denitrifying organisms affects the growth of MetH-dependent environmental isolates from the rhizosphere, expanding on previous work showing this effect in engineered organisms. A clear implication is that soil denitrifiers might have the capacity to disrupt microbial communities that help support plant health. Cobalamin, along with other structurally related cobamides, is widely recognized as a mediator of microbial interactions (25, 27, 77), and it seems likely that vitamin auxotrophs like *Pseudoxanthomonas* sp. Root65 acquire cobalamin from co-occurring microbes. Interestingly, cobalamin showed variable capacity to rescue the growth of Δ*metE* mutants and sensitive rhizosphere isolates (Fig. 1B, 3B, and 4B), a result that could be explained by differences between exogenous and physiological N_2_O concentrations, the capacity to uptake cobalamin from agar plates, or underlying cobamide preferences (78). In contrast, methionine reliably rescued these sensitive organisms and might be sourced from either plants or co-occurring microbes. Plants do not have the capacity to synthesize cobalamin and therefore exclusively produce methionine via a MetE homolog. This amino acid is central to many metabolic processes in plants (79) and has been detected in root exudates (80, 81). Periodic nitrous oxide emissions have been correlated with conditions common to the rhizosphere, such as high nitrate and organic carbon (38, 40, 82), and with increased soil moisture as may occur following rain or irrigation events (9, 39). During these day-to-week long bursts (44), N_2_O can reach hundreds of micromolar (as summarized in references 83, 84). While more studies will be needed to understand how cobalamin and L-methionine are exchanged in the rhizosphere, our results offer the testable hypotheses that some microbes may experience a heightened reliance on plant sources of L-methionine during such hot moments of N_2_O production.

In addition to implications for microbe–microbe and plant–microbe interactions, our results reframe enzymes in both the methionine synthesis and denitrification pathways as playing both protective and biochemical roles, adding to growing evidence that detoxification of nitrogen cycle intermediates may serve an underappreciated role in biogeochemical cycling. MetE is often considered a strategic backup enzyme that allows growth in the absence of cobalamin. Our work, along with that of others (20, 21), now presents substantial support for the idea that MetE may represent a means of protection from N_2_O. These findings also provide perspective on the newly discovered Mes enzymes, one of which (MesD) we show is also insensitive to N_2_O (Fig. 4B and C). Additionally, NosZ, the terminal nitrous oxide reductase, is appreciated as the only biological loss process for N_2_O and has been historically studied as one component of the energy-conserving denitrification apparatus. However, this enzyme occurs in three distinct forms (85), and some or all of these versions may serve a secondary role in detoxification. A similar role for the nitric oxide reductase has been demonstrated in relation to NO, the toxic intermediate that precedes N_2_O in canonical denitrification. Co-culture studies in *P. aeruginosa* using engineered NO producers and consumers have shown that the need to detoxify NO shapes spatial organization in biofilms (2). More recent work has shown that a set of flavohemoglobin proteins (Fhp) which detoxify NO to N_2_O independent of energy conservation may represent an underappreciated source of N_2_O (73).

While nitric oxide and its derivatives react with and damage a range of biological molecules, N_2_O toxicity is much more specific and instead relies on the high reactivity of the unique Co(I) state potentiated by some cobalamin-dependent enzymes. Our findings predict that negative selection against organisms dependent on Co(I)-mediated processes may be occurring in environments experiencing continuous and/or periodically high exposure to N_2_O. Some evidence for this recently emerged from bioreactor studies where high levels of N_2_O appeared to dictate both NosZ usage and cobalamin biosynthetic capacity (86–88). In addition to natural and agricultural soils, other plausible locations where N_2_O reaches high concentrations include wastewater treatment facilities (13), marine oxygen minimum zones (10, 11), and pulmonary infections (89). It is also notable that while we focus on the methionine synthase, other microbial enzymes that utilize cobalamin-derived Co(I) in catalysis, including those involved in methanogenesis (23), reductive dechlorination (22), and mercury methylation (24), have also been shown to be inhibited by elevated concentrations of N_2_O. Some of these studies report *in vivo* inhibitory constants (*K_I_*) on the order of 10 to 100 µM (22, 23), approximately equal to concentrations observed during hot moments in the environment (83). To our knowledge, no studies have determined these constants for MetH. Despite this, we observed growth inhibition of sensitive strains at high micromolar to low millimolar concentrations, consistent with previous investigations (21). Further studies that probe the physiological effects of a range of N_2_O concentrations on sensitive microbes and consider spatial structuring and metabolic exchange in microbial communities will be needed to better understand the consequences of N_2_O production at the microscale. However, the work here clearly shows that N_2_O is widely toxic to MetH-reliant organisms and that endogenous levels of the gas can affect microbial growth. We expect future studies to continue to reveal more examples of the ways this climate-active gas impacts microbial community composition.

## MATERIALS AND METHODS

### Bacterial strains and construction of deletion mutants

Strains used in this study can be found in Table S1. Plasmids and primers can be found in Table S2. Markerless, in-frame deletions of *metE* and *nosZ* in *P. aeruginosa* were constructed by allelic exchange. Up and downstream regions flanking the gene of interest (800–1,000 bps) were inserted into the pMQ30 vector by Gibson Assembly (90) and introduced into *P. aeruginosa* via triparental conjugation. Merodiploids were selected for on Vogel–Bonner minimal medium with 50 µg/mL gentamicin (91, 92) with counter-selection on Luria-Bertani (LB) + 5% sucrose (by weight). Mutants were confirmed by PCR. Gentamicin-resistant markers were integrated into the *att*Tn7 site using the pUC18-mini-Tn7T-Gm delivered via conjugation (92). For complementation, PA14 *metE,* in addition to 400–500 bp up and downstream of the gene, was inserted into the *att*Tn7 site as described above. For all experiments, strains were first struck on LB agar and a single colony was used to inoculate LB broth, which was subsequently used to inoculate a defined medium unless otherwise noted (pH 7.5, 37 mM succinate, 0.68 mM CaCl_2_, 0.41 mM MgSO_4_, 4.7 mM KH_2_PO_4_, 2.3 mM K_2_HPO_4_, 20 mM 3-N-morpholinopropanesulfonic acid, Aquil trace metals with copper omitted [93], N-source for each experiment indicated below). All chemicals were obtained from Sigma except for methylcobalamin, which was obtained from Cayman Chemical at a purity of ≥98% (Cat. 29113). *P. aeruginosa* strains were routinely grown at 37°C; all others were maintained at 30°C in vented tissue culture flasks or 96-well plates with lids unless otherwise noted.

### Bacterial growth under exogenous nitrous oxide

Prior to assaying growth, strains were aerobically passaged four times on defined medium modified from above with three carbon sources: 8.22 mM glucose, 12.33 mM succinate, 16.44 mM pyruvate; 20 mM NH_4_Cl, 250 nM CuSO_4_, and amino acids assembled to match concentrations of 1× minimal essential medium (MEM) essential and non-essential amino acid solutions (Gibco 11130051 and 11140050) without L-methionine and with vitamins provided as the 0.5× MEM vitamin solution (Gibco 11120052, no cobalamin). Cultures were then serially diluted and 5 µL of the diluent was spotted onto defined media plates with or without 250 µM L-methionine or 50 nM cobalamin (only isolates determined to be sensitive to N_2_O were tested for cobalamin rescue). Plates were incubated at 20°C in the dark in a sealed box (BD GasPak EZ Container) flushed continuously (300 mL/min) with 50% N_2_O (Linde 99.5% purity) or N_2_; see Fig. S1A. After 96 h, plates were imaged and compared to plates grown in parallel under an unmodified atmosphere. One of the tested At-RSPHERE isolates, *Pseudoxanthomonas* sp. Root65 was determined to be a cobalamin auxotroph (Fig. S7) and required the addition of cobalamin to facilitate routine growth.

### *P. aeruginosa* denitrifying growth

To initiate denitrification, *P. aeruginosa* cultures were grown statically in vented tissue culture flasks in an anoxic chamber filled with 5% H_2_, 95% N_2_ headspace (Coy) and serially passaged four times on defined media containing 20 mM KNO_3_ and 37 mM succinate. *P. aeruginosa* Δ*metE* cultures were supplemented with 250 µM L-methionine during this conditioning period. Prior to experiments, cells were pelleted (6,800 × *g* for 15 min), washed in NO_3_^−^ and L-methionine free media, and inoculated into experimental media at a starting OD500 = 0.01. Growth was measured as OD500 in 96-well plates with lids by a BioTek Epoch 2 or Synergy HTX inside the anaerobic chamber with 10 s of shaking prior to each hourly OD reading. Experiments under oxic conditions were established as above, except cultures were pre-cultured under ambient air with shaking at 200 RPM and continuous shaking for plate reader experiments.

### Measurement of N_2_O production and consumption from *P. aeruginosa* cultures

Detailed experimental procedures for N_2_O measurements are provided in the Supplemental methods. Briefly, amperometric N_2_O microsensors (Unisense) with piercing needle probes were used to measure N_2_O in all experiments. Measurements of N_2_O from denitrifying cultures were either conducted in 10 mL sealed glass serum vials (Fig. 2B and C; Fig. S2, S3, and S4A), tissue culture flasks open to the atmosphere (Fig. S8), or 96-well plates open to the atmosphere (Fig. S4B). With the exception of Fig. 2B, where N_2_O was measured in the headspace, N_2_O was measured in the dissolved phase.

### Identification of methionine synthase repertoires in rhizosphere isolates and phylogenomic reconstruction

Gapmind (94) was used to identify methionine synthase candidates in At-RSPHERE genomes (BioProject PRJNA297942) (66). The parameters used to annotate and classify these genomes are provided in the Supplemental methods. A phylogenomic tree was assembled using GToTree v1.8.6 (95) with the prepackaged 74 single-copy gene set for bacteria. Target genes were identified in At-RSPHERE protein fasta files using HMMER3 v3.4 (96). Genes were aligned with muscle v5.1.osx64 (97) and trimmed with trimAl v1.4.rev15 (98). Phylogenetic estimation of the concatenated alignments was performed by FastTree2 v2.1.11 (99) and annotation data from Gapmind was appended using treeio v1.26.0 (100). The tree in Fig. 4A was rooted at the midpoint using phangorn v2.12.1 (101) and visualized using ggtree v3.10.1 (102). See Fig. S6A for a more detailed tree.

### Co-culturing conditions

For oxic *P. aeruginosa*-*P. aeruginosa* co-cultures, gentamicin-resistant *P. aeruginosa* (Δ*metE_gentR_*) and gentamicin-sensitive *P. aeruginosa* Δ*nosZ* strains were first passaged four times on a defined medium (20 mM NO_3_^−^ as the only N source). After which they were harvested by centrifugation (10,000 × *g* for 2 min), washed in N-free medium, mixed 1:1, and inoculated at an initial OD500 = 0.01 in medium containing 20 mM NO_3_^−^. The relative abundance of Δ*metE_gentR_* was quantified by selective plating on LB + gentamicin (50 µg/mL) after 24 h of shaking growth at 37°C under ambient air (Fig. 3D). Anoxic co-cultures were established as described above except: *P. aeruginosa* cells were transferred under anoxic conditions with 250 µM L-methionine and 20 mM NO_3_^−^ four times prior to co-culture setup to establish denitrification. Co-cultures were given 10 pM cobalamin and were grown statically at 37°C for 24 h before the relative abundance of the gentamicin-resistant strain was quantified.

Co-cultures with *Pseudoxanthomonas* sp. Root65 were conducted statically under hypoxic conditions. *P. aeruginosa* WT was pre-grown without oxygen, while *Pseudoxanthomonas* sp. Root65 was pre-grown under ambient air and supplemented with 250 µM L-methionine. After four independent *P. aeruginosa* WT and *Pseudoxanthomonas* sp. Root65 transfers, cultures were washed and mixed 1:1 at starting OD500 = 0.01 in a medium containing 100 pM cobalamin (approximately the EC50 for *Pseudoxanthomonas* sp. Root65; see Fig. S7) and 250 nM Cu and 0, 5, 10, or 20 mM NO_3_^−^ (balanced to 20 mM total N with NH_4_^+^) and grown for 24 h. For CFU counts, co-cultures were plated on LB and LB + gentamicin. *Pseudoxanthomonas* sp. Root65 (which is naturally resistant to gentamicin) was enumerated after 72 h. *P. aeruginosa* was enumerated on the LB plates without antibiotics at 24 h before any *Pseudoxanthomonas* sp. Root65 colonies form (see Fig. S9B and D).

### DNA extraction and qPCR

Genomic DNA was extracted from 1 mL of co-culture using the DNeasy Blood & Tissue Kit (Qiagen) and quantified using a Qubit Fluorometer (Invitrogen). qPCRs were conducted with the iTaq Universal SYBR Green Supermix (Bio Rad) on the CFX96 Real-Time PCR Detection System (Bio Rad) using 500 pg of co-culture genomic DNA and 500 nM species-specific primers (see Table S2 for sequences) per assay. Sample and reaction details, and cycling parameters are provided in the Supplemental methods. Additional qPCRs with off-target primer-DNA pairings were conducted to control for cross-amplification (see Fig. S10 and Supplemental methods).

## SUPPLEMENTAL MATERIAL

Supplemental Material mbio.02699-25-s0001.pdf Supplemental methods and Figures S1-S10.

Table S1 mbio.02699-25-s0002.xlsx Bacterial strains.

Table S2 mbio.02699-25-s0003.xlsx Plasmids and primers.

Table S3 mbio.02699-25-s0004.xlsx Methionine synthase genome hits.

ASM does not own the copyrights to Supplemental Material that may be linked to, or accessed through, an article. The authors have granted ASM a non-exclusive, world-wide license to publish the Supplemental Material files. Please contact the corresponding author directly for reuse.
