# Supplementary material for: Nitrous oxide produced by denitrifying pseudomonads inhibits the growth of rhizosphere bacteria by inactivating the cobalamin-dependent methionine synthase
Source: mBio. 2026 Mar 4;17(4):e02699-25. doi: 10.1128/mbio.02699-25 (PMC13059711; doi:10.1128/mbio.02699-25)
Supplement: Supplemental Material — Supplemental methods and Figures S1-S10. [file mbio.02699-25-s0001.pdf]

SUPPLEMENTAL MATERIAL

Supplemental Figures

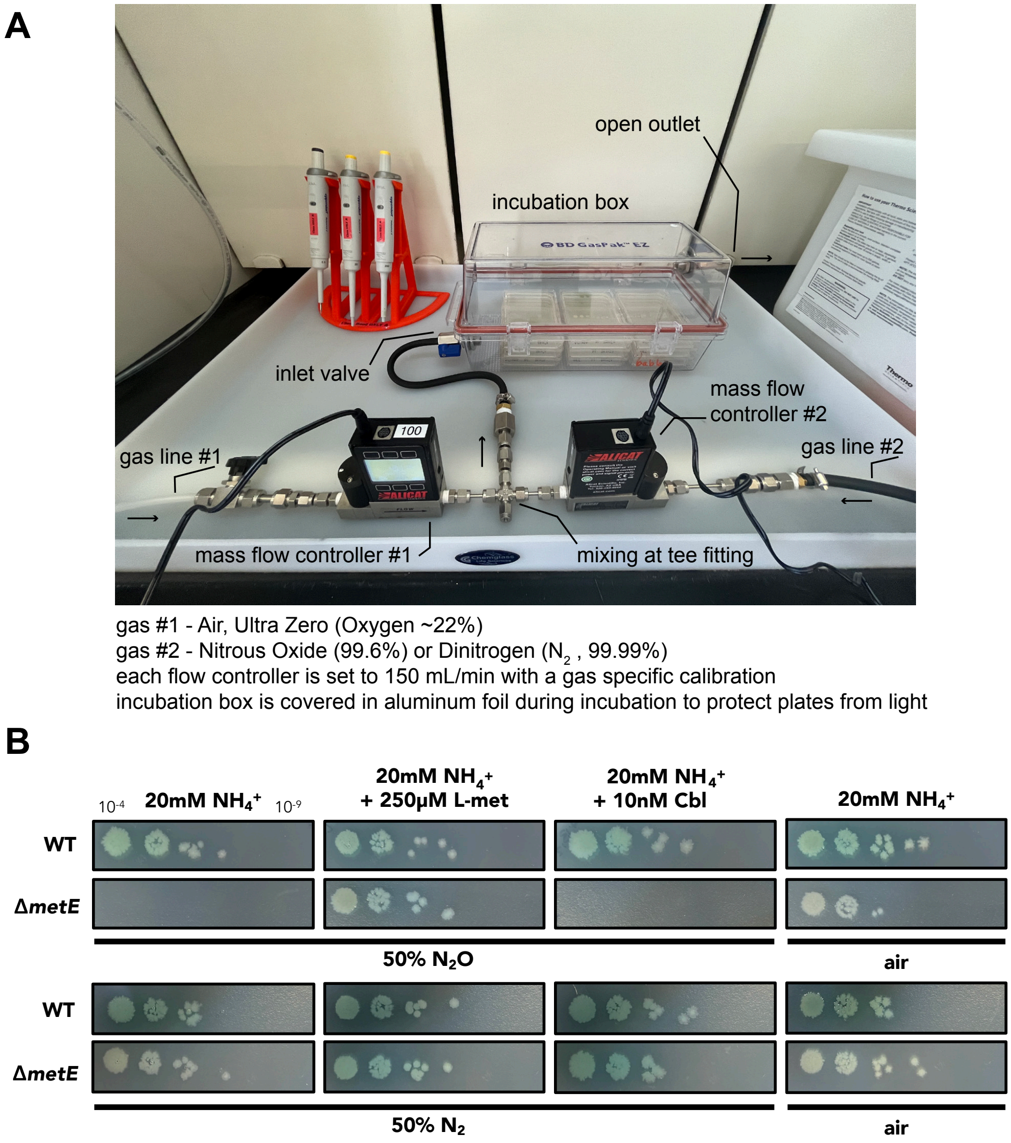

Fig S1 Incubation of agar plates under modified atmospheres. (A) Setup of the mechanism for delivery of a modified atmosphere to bacteria grown on agar plates. The flow rate of gases used in an experiment are set by two mass flow controllers (Alicat

Scientific) calibrated for each gas. By matching the flow rate of each gas, mixing occurs in a simple tee fitting before being delivered to the sealed incubation box, resulting in approximately 50% of the desired gas (N_2O or N_2) mixed with air. (B) The growth of *P. aeruginosa* Δ*metE* is inhibited under an atmosphere of 50% N_2O but not under 50% N_2. Plates were incubated under their respective modified atmosphere for 24 hours at 37 ˚C prior to imaging.

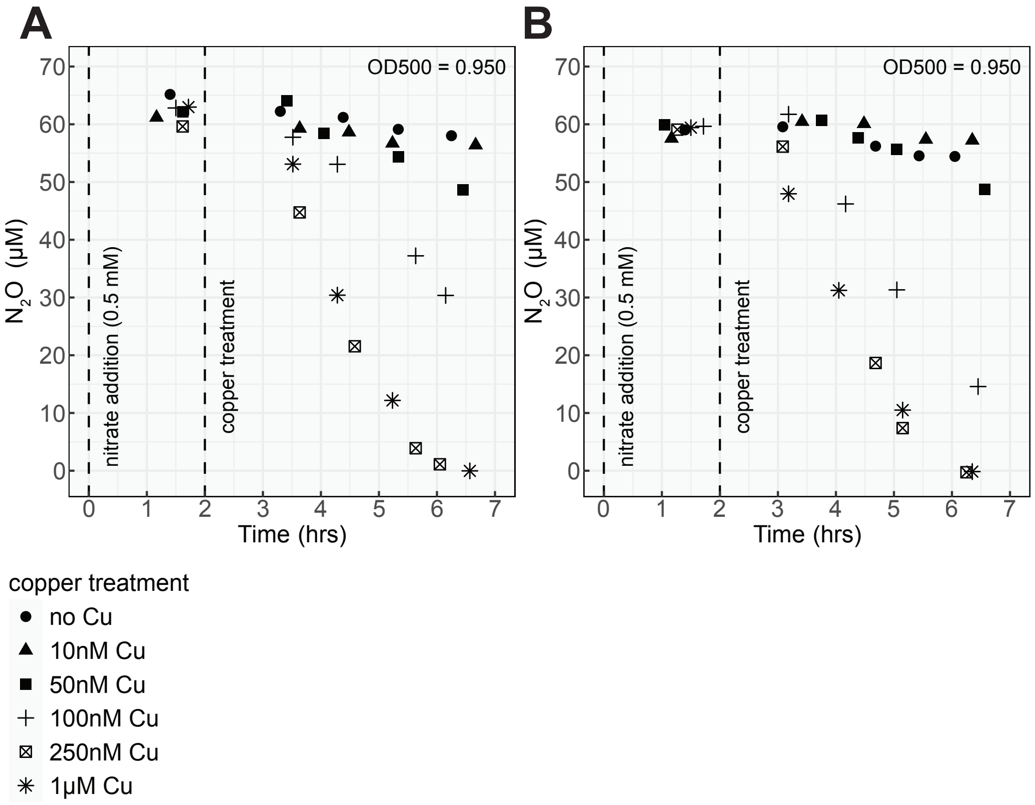

Fig S2 Copper induces an increase in the rate of N_2O consumption from the headspace of *P. aeruginosa* WT cell suspensions. *P. aeruginosa* WT cells harvested during exponential phase of anaerobic growth were provided 0.5 mM nitrate in a sealed vial and were incubated at 20˚C for approximately 1 hour, during which they accumulated N_2O in the headspace. The addition of copper sulfate solutions of increasing concentrations led to increased consumption of N_2O from the headspace. The two panels (A and B) depict the data from two biological replicates from a single experiment.

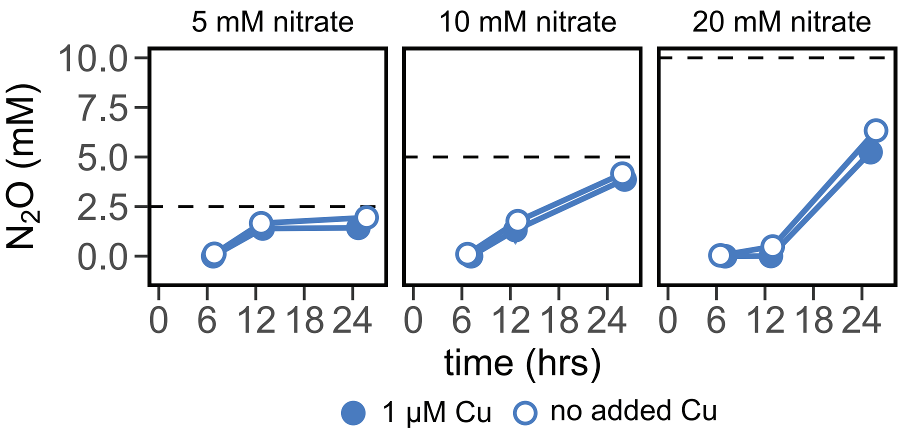

Fig S3 Nitrate concentration influences N_2O accumulation in anoxic denitrifying *Pseudomonas aeruginosa* cultures. This experiment replicates the experiment presented in Fig. 2C. Points represent the mean of 2 biological replicates. Error bars, which may be obscured by the data-point, depict ±SD. Horizontal dashed lines indicate the theoretical maximum yield of N_2O that can be generated from a given NO_3^- concentration.

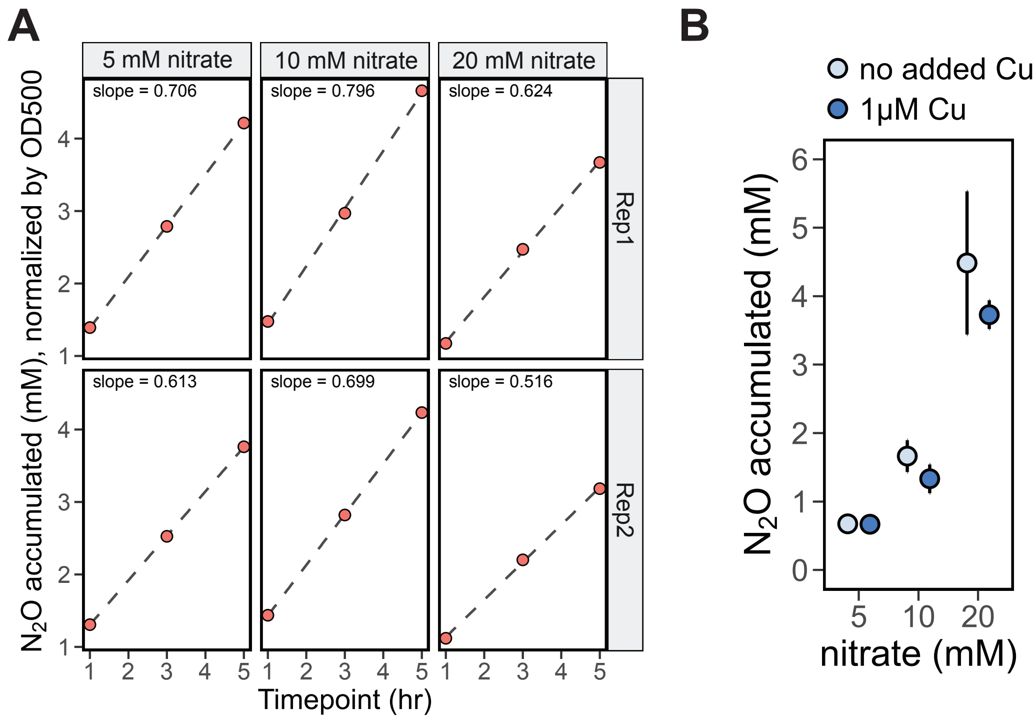

Fig S4 The effect of nitrate availability on the rate of N_2O production and accumulation in *P. aeruginosa* WT cultures. (A) The rate of N_2O accumulation in exponential phase *P. aeruginosa* WT cells is largely insensitive to the different nitrate concentrations tested (5, 10, 20 mM). A slight, but not significant, decrease is observed under the highest concentration. A Kruskal-Wallis test to compare the slope coefficients across the three nitrate conditions found no significant difference between groups (Kruskal-Wallis chi-squared = 2.5714, df = 2, p-value = 0.2765) (B) Nitrate determines the amount of N_2O dissolved in 2mL of "open" (i.e. not sealed vial) *P. aeruginosa* cultures after 24 hours of growth at 37˚C. Copper appears to have a marginal, but not significant effect, on N_2O accumulation, consistent with results in Fig. 2C. Log-transformed mM values were compared across nitrate and copper conditions using two-way ANOVA (nitrate: $F_{2,12}$ = 231.55, p < 0.0001; copper: $F_{1,12}$ = 3.73, p = 0.077; and interaction: $F_{2,12}$ = 0.91, p = 0.429) with Tukey-adjusted post-hoc tests showing no significant effect of copper at each nitrate level (5 mM: estimate = 0.006, SE = 0.119, p = 0.960; 10 mM: estimate = 0.225, SE = 0.119, p = 0.083; 20 mM: estimate = 0.166, SE = 0.119, p = 0.187). Points represent the mean of 3 biological replicates ± SD.

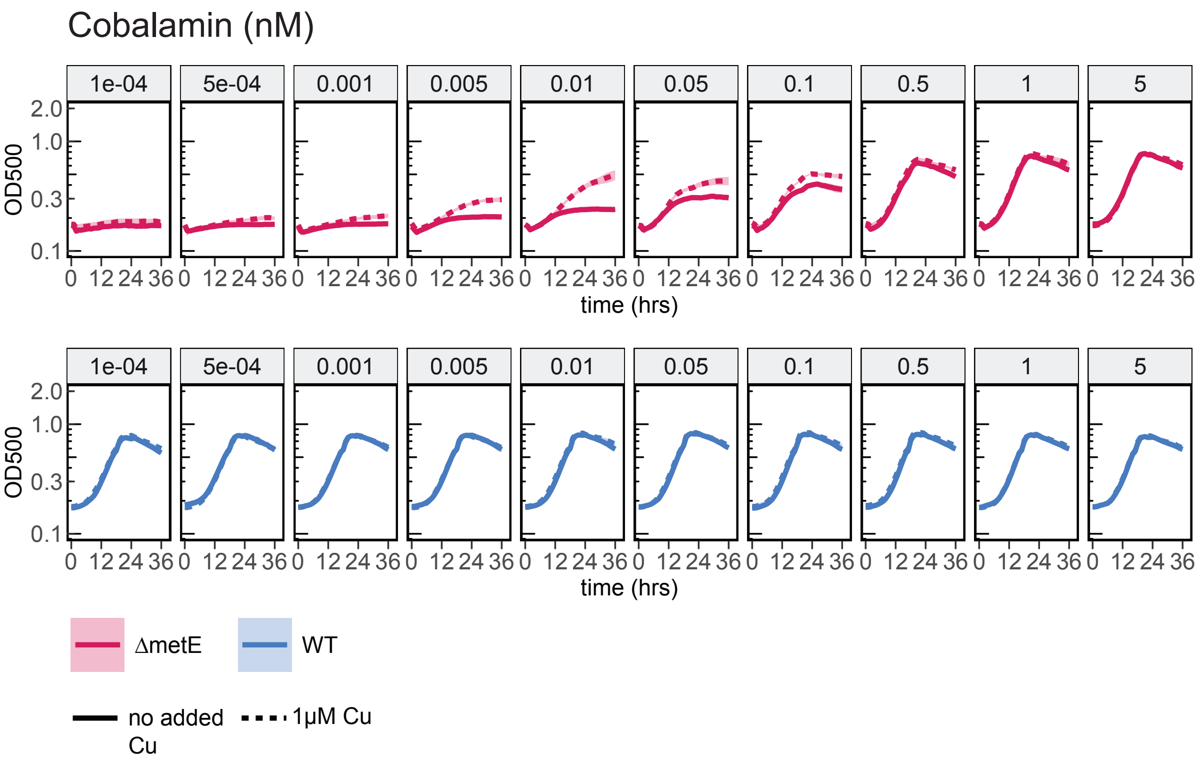

Fig. S5 Exogenous supplementation of cobalamin rescues the growth of *Pseudomonas aeruginosa ΔmetE* during anoxic growth. Data presented in Fig. 3B were selected to present from this larger dataset (1 pM, 10 pM, and 1 nM, also plotted here). Lines represent the mean of three biological replicates ± SD (shaded area).

A

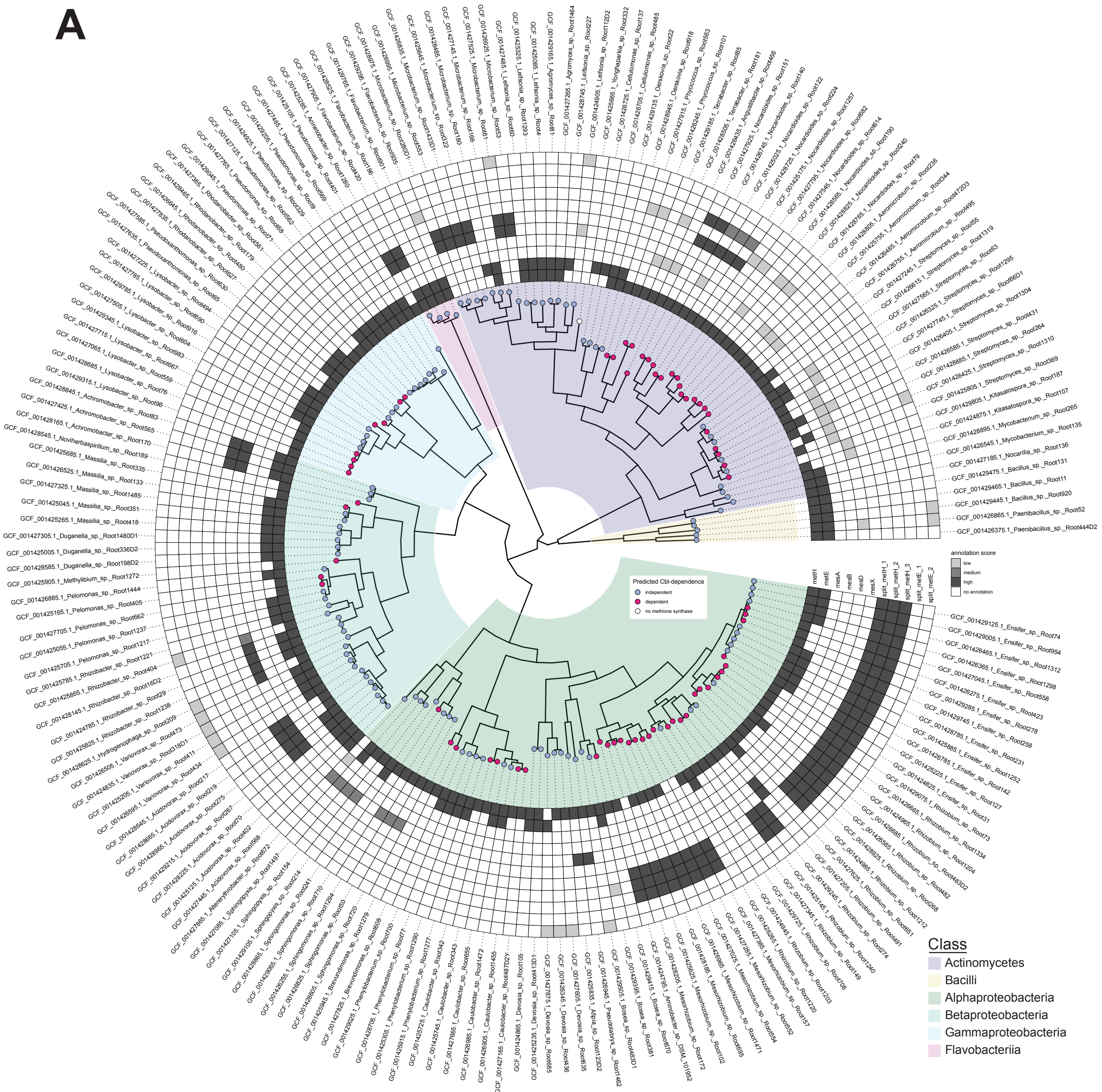

Predicted Cbl-dependence
independent
dependent
no methione synthase
metH
metE
mesA
mesB
mesD
mesX
split_metH_1
split_metH_2
split_metH_3
split_metE_1
split_metE_2
annotation score
low
medium
high
no annotation
Class
Actinomycetes
Bacilli
Alphaproteobacteria
Betaproteobacteria
Gammaproteobacteria
Flavobacteriia

B

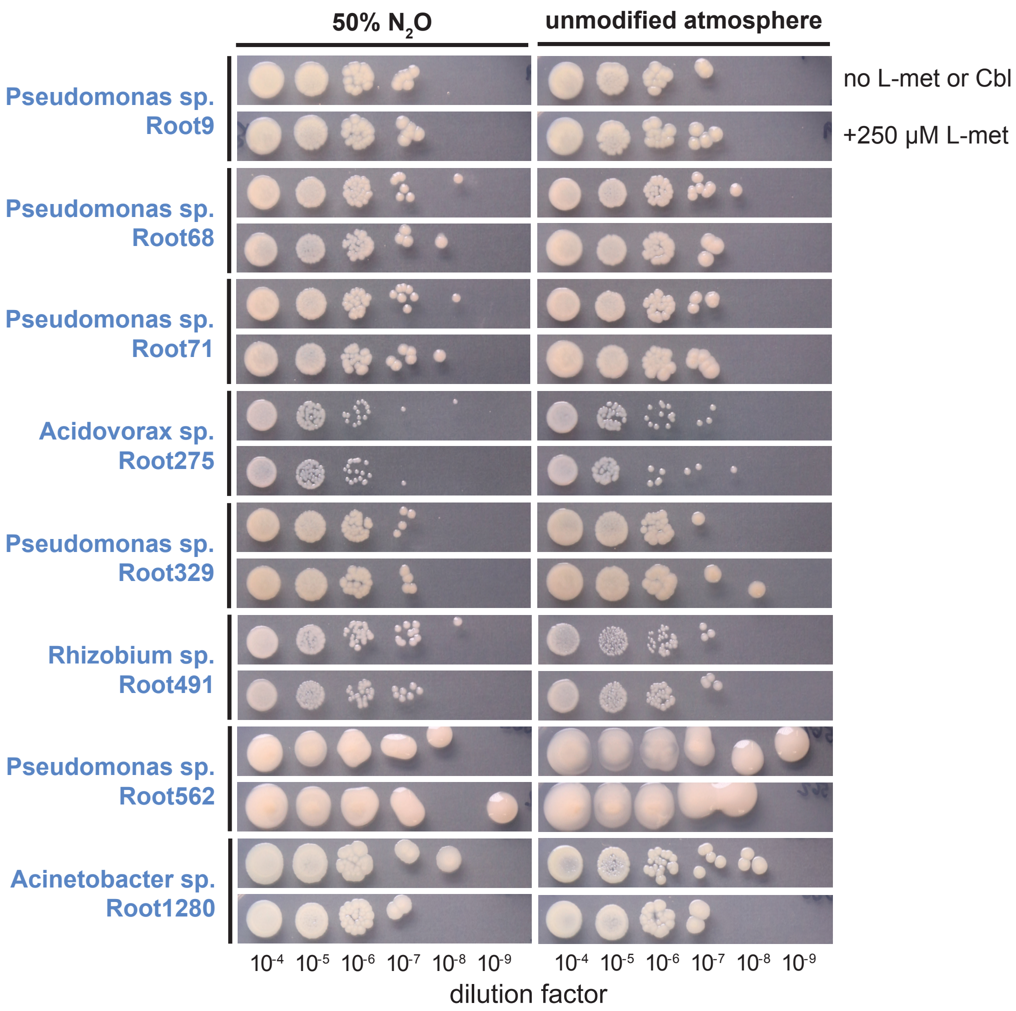

50% N_2O
unmodified atmosphere
no L-met or Cbl
+250 μM L-met
Pseudomonas sp. Root9
Pseudomonas sp. Root68
Pseudomonas sp. Root71
Acidovorax sp. Root275
Pseudomonas sp. Root329
Rhizobium sp. Root491
Pseudomonas sp. Root562
Acinetobacter sp. Root1280
10^{-4} 10^{-5} 10^{-6} 10^{-7} 10^{-8} 10^{-9}
10^{-4} 10^{-5} 10^{-6} 10^{-7} 10^{-8} 10^{-9}
dilution factor

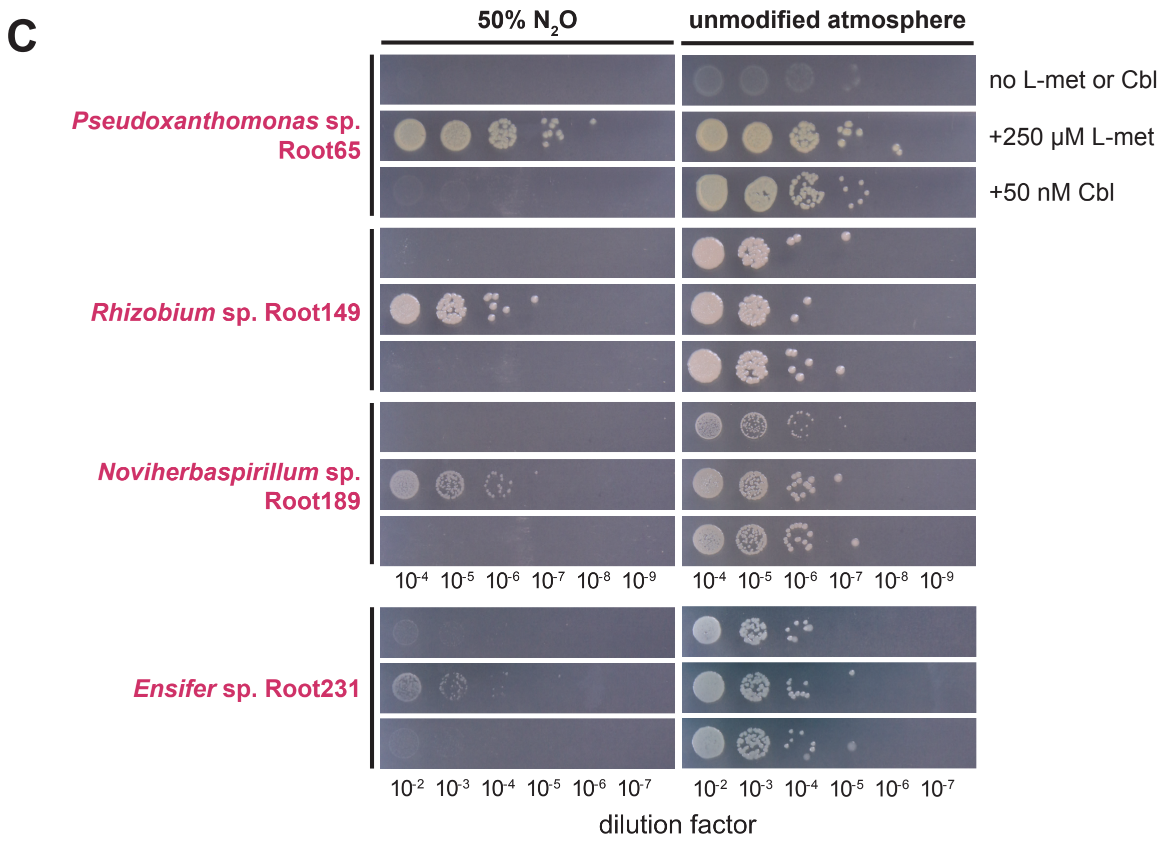

Fig S6 The At-RSPHERE culture collection hosts isolates reliant on cobalamin dependent methionine synthases determined to be sensitive to N_2O inhibition. (A) Complete phylogenomic tree of the At-RSPHERE culture collection with methionine synthase annotations. A version of Fig. 4A with tree tips labeled with genome identifiers and annotations. See Table S3 for more information on annotations. (B) Eight strains tested for growth under 50% N_2O were determined to be tolerant to the gas and (C) four strains were determined to experience growth inhibition. The 10^{-5} dilutions (or 10^{-3} in the case of Root231) seen above were used to assemble composite images in Fig. 4B.

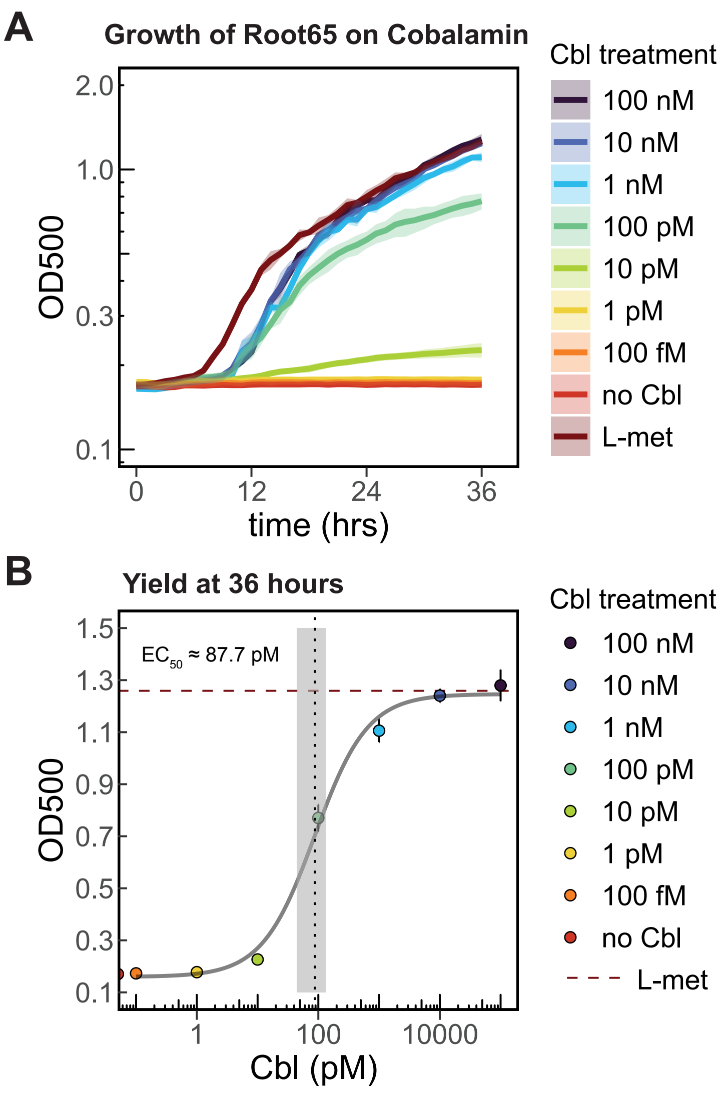

Fig S7 ***Pseudoxanthomonas*** **sp. Root65 is a cobalamin auxotroph**. A dose-response relationship between growth of *Pseudoxanthomonas* sp. Root65 and cobalamin concentration was observed. (A) Root65 strictly requires cobalamin or L-methionine for growth. Lines are the mean of 3 biological replicates ± SD (shaded area). (B) The yield (OD500) at 36 hours of growth was used to determine the cobalamin concentration resulting in half-maximal growth (EC_{50}). A four-parameter logistic regression model (LL.4) was fit to OD500 measurements across the range cobalamin concentrations (0 to 100nM), excluding the L-methionine control (depicted as a horizontal dashed maroon line). The fitted curve is shown in grey, with a shaded box region indicating the 95% confidence interval around the estimated EC_{50}. The dotted vertical line marks the EC_{50} (approximately 87.7 pM). Points represent the mean OD500 at 36 hours ± SD.

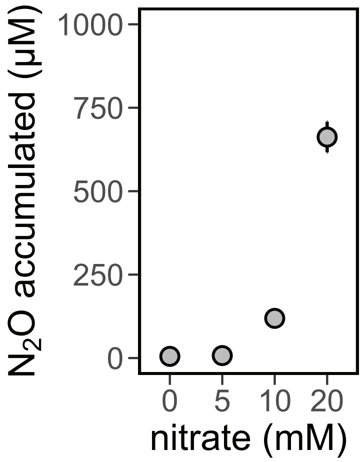

Fig S8 N_2O accumulates in hypoxic *P. aeruginosa* WT – Root65 co-cultures. After 24 hours of growth, co-cultures with high nitrate accumulated N_2O despite being grown under ambient air with 250 nM Cu. Points represent the mean of 3 biological replicates ± SD.

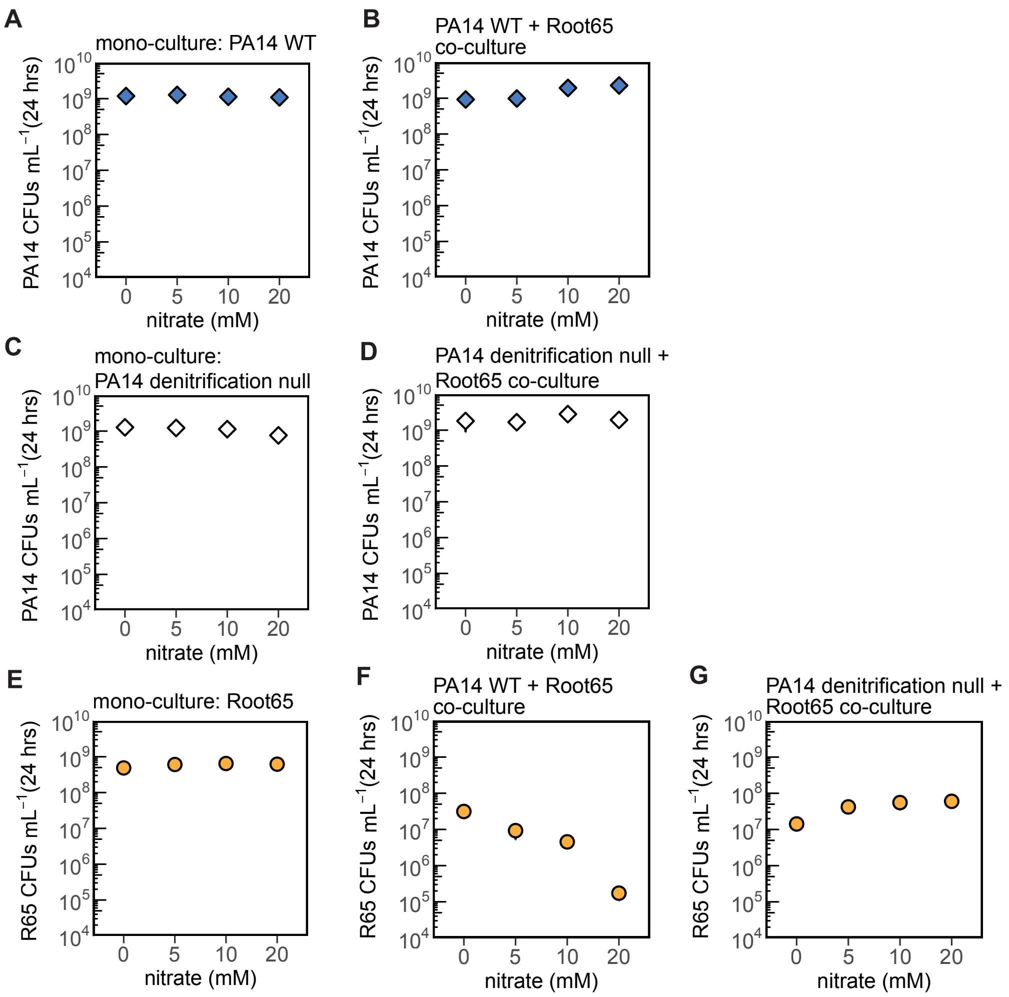

Fig S9 The yields of *Pseudoxanthomonas* sp. Root65 (R65) and *Pseudomonas aeruginosa* (PA14) WT and denitrification null (ΔnarGHJI, ΔnirS, ΔnorBC, ΔnosZ, Δfhp) strains are largely unaffected by nitrogen source in mono-culture. (A,C,E) Strains were grown in mono-culture in the same conditions used to set up to co-cultures in Fig. 5B. After a 24-hour incubation at 30˚C growth was quantified by counting CFUs. (B,D) *P. aeruginosa* strains reach similar yields in co-culture with *Pseudoxanthomonas sp. Root65*. Following the co-culture presented in Fig. 5B, cells were plated on LB plates without antibiotics and CFUs were counted after 24 hours of growth. These colonies were determined to be *P. aeruginosa* due to their colony morphology and because *Pseudoxanthomonas* sp. Root65 consistently requires at least 48 hours to produce countable colonies. (D,G) *Pseudoxanthomonas* sp. Root65 abundances from co-cultures are replotted here for comparison (see Fig. 5B). All CFUs mL^{-1} values are presented as the mean of three biological replicates, with ± 1 SD error bars which may be hidden by points.

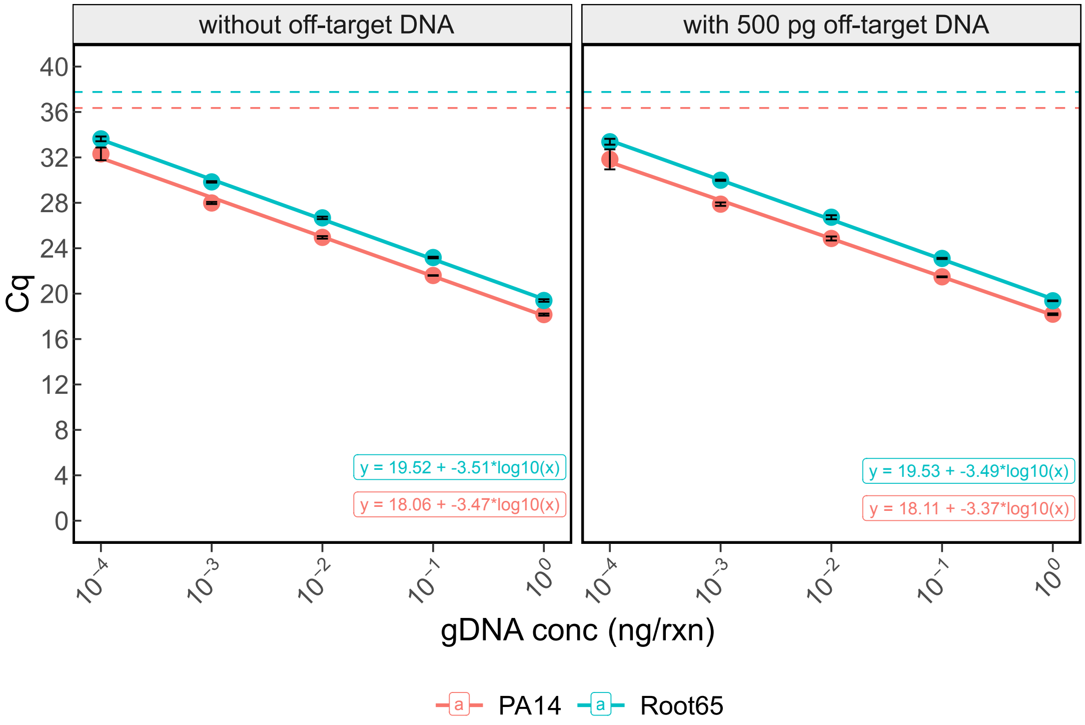

Fig S10 Species-specific primers amplifying the gene *secY* do not exhibit significant cross-reactivity in the presence of off-target DNA from the opposite species. Standard curves from qPCR assays with *P. aeruginosa* (red) and Root65 (blue) genomic DNA (gDNA) and species-specific primer pairs (see table S2), with and without the addition of off-target gDNA from the opposite species (500 pg). Points show the mean Cq of three replicates ± SD; regression equations are displayed. Linear regression analysis with log-transformed Cq values revealed no significant difference in amplification slopes between assays with and without off-target DNA (*P. aeruginosa*: $p = 0.699$; Root65: $p = 0.972$ for interaction terms), demonstrating robust quantification without significant cross-reactivity at these DNA concentrations. Horizontal dashed lines indicate the mean Cq for qPCR assays amplifying 1 ng gDNA with a primer pair designed for the opposite species (PA14 gDNA with Root65 primers (red) and Root65 gDNA with PA14 primers (blue)). Off target Cqs are much higher than any Cq used for quantification, further providing support that cross-amplification had a negligible effect on species-specific quantification.

Supplemental Methods

Analytical methods for measurements of N_2O

Nitrous oxide was detected using amperometric sensors (Unisense) integrated into piercing needles. Two sets of sensors were used, the standard needle piercing sensor with dynamic range of 0.1 µM – 50 µM was used for initial experiments with low added nitrate (Fig. 2B, S2), and the High Range sensor, with a dynamic range of 1 µM – 4mM was needed to tolerate higher concentrations of N_2O accumulated in experiments with higher nitrate (Fig. 2C, S3, S4, S8). A two-point standard curve of dissolved N_2O was used to convert milli-volt measurements to molarity units. It is notable that these sensors have known interferences with NO, which may contribute to our results. However, our general observation of stoichiometric conversion of nitrate to N_2O that remains stable over time (Fig. 2C, S3), suggests that these measurements reflect realistic N_2O concentrations. In all experiments except for Fig. 2B, N_2O was measured in the dissolved phase and the concentrations (in µM) detected are reported. In Fig 2B the concentration of N_2O was measured in the headspace of a sealed vial over time. Here N_2O is reported as total micromoles/vessel, calculated using the Henry's law constant for N_2O (2.4×10^{-4} mol /m^3 Pa) according to the equation:

$$H^{cp}RT = C_a/C_g$$

Where H^{cp} is Henry's constant, R is the universal gas constant, T is temperature, C_a is the aqueous N_2O concentration (µM) and C_g is the gas phase concentration (µM).

Determination of N_2O production and consumption in response to copper

For initial comparisons of the short-term effects of Cu on WT and Δ*nosZ* strains (Fig. 2B, S2) denitrifying anoxic cultures were resuspended to an OD500 of 1.0 in NO_3^- and Cu free media in vials. All media was prepared in acid cleaned polycarbonate bottles to avoid background Cu. Nitrate (0.5 mM) was added immediately before the start of experiments. Incubations were conducted at approximately 20°C. At 2 hours, $CuSO_4$ was added via syringe. In the experiment presented in Fig. 2B, N_2O was measured in approximately 7.25 mL of headspace and converted to µmols per vessel as described above. In all others (Fig 2C, S3, S4A) glass serum vials were filled without headspace and N_2O was detected in the liquid after thorough vortexing. A range of Cu concentrations were tested in Fig. S2 to determine the amount required for NosZ activity (10 nM, 50 nM, 100 nM, 250 nM, 1 μM).

For determination of the effect of copper concentrations on N_2O accumulations over time (Fig. 2C), *P. aeruginosa* WT cells were grown without added Cu in the absence of oxygen for a period of 4 transfers in defined media. In an anoxic chamber, experimental cultures were inoculated to a starting OD500 of 0.01, indicated NO_3^- amounts were added, and solutions

were aliquoted into acid washed 10 mL glass vials. Sealed vials were incubated at 37˚C. At the timepoints indicated, sacrificial vials were pulled from the incubator and anoxic chamber, vortexed, and measured by piercing the septa with the sensors needle. Sensors were given 3 minutes to equilibrate in each vial after which the reading was recorded. In between the measurement of each vial the sensor was washed with 70% ethanol, rinsed with water, and allowed to equilibrate with air (approximately 3 minutes) before measuring the next sample. At each timepoint the order in which samples were measured was randomized. All vials were kept at 37˚C before and during measurement.

Estimation of N_2O production rates from exponential phase *P. aeruginosa* cell suspensions

To determine rates of N_2O production (Fig. S4A), *P. aeruginosa* WT cells were grown anaerobically for 17 hours on 20 mM nitrate before being harvested via centrifugation, washed out of nitrate, and resuspended to an OD500 = 0.2 in a medium without any nitrogen source or added copper. Cells were incubated for 1 hour at 37˚C to deplete intracellular nitrate. Then, 5, 10, or 20 mM NO_3^- (balanced to 20 mM total N with NH_4^+ when needed) was added. 8.5 mL of these cell suspensions were aliquoted into vials with minimal headspace and incubated at approximately 20˚C. The N_2O produced in these vials was measured at 1, 3 and 5 hours after sealing vials. Vials were vortexed prior to measurement and sampling was destructive, meaning timepoints come from discrete vials. Replicates are from two independent experiments. N_2O concentrations were normalized by initial vial OD500 and were fit to a linear regression model of N_2O (mM) as a function of time (hours). The slope coefficient was extracted and used to compare the rate of N_2O production across NO_3^- concentrations.

Measurement of N_2O from open cultures

In Fig 2BC we measure N_2O accumulation in sealed (“closed”) culture vessels. However, subsequent experiments where we investigate the effect of N_2O accumulation on sensitive strain growth were conducted in culture vessels where we may expect exchange with the atmosphere (i.e. in 96-well plates with standard covers and 20mL culture flasks with vented caps). Therefore, we sought to measure N_2O accumulation in such an “open” system (Fig. S4B, S8). *P. aeruginosa* WT cells conditioned to denitrify were inoculated in 2 mL of media in 96 deep-well plates with 5, 10, and 20 mM NO_3^- and grown for 24 hours anaerobically. Plates were covered with aluminum plate foil during growth. Probes were pierced through the foil to take N_2O measurements in the liquid phase (see Fig. S4B). Additionally, N_2O from anoxic *P. aeruginosa* – Root65 co-cultures grown under static oxic

conditions were measured. These cultures were grown in 5 mL of media in 25 mL culture flasks with vented caps in the presence of 250 nM Cu (see Fig S8).

Parameters used to annotate At-RSPHERE Genomes

The script gapsearch.pl, provided by Gapmind, was used to search for potential proteins in each At-RSPHERE genome against a curated set of methionine biosynthesis proteins (see Table S3 for definitions of protein annotations). Candidates were removed if the bitscore of the methionine synthase annotation was lower than that of a divergent protein (as provided by gaprevsearch.pl). In the case of multiple hits, the annotation with the highest Gapmind score, or in the case of a tie, bitscore was selected. A genome was classified as cobalamin-independent if it encodes any *metE*, *mesD*, or either part of the split *metE* annotation regardless of *metH* presence. A genome was classified as cobalamin-dependent if it encoded any *metH*, *mesA*, *mesB*, or all three split *metH* parts and lacked any cobalamin-independent alternative. As expected, no MesC annotations (archaeal) were found in the bacterial dataset.

Determination of cobalamin requirement for *Pseudoxanthomonas* sp. Root65

During growth on defined media plates, we noticed that *Pseudoxanthomonas* sp. Root65 failed to produce robust colonies without the exogenous supply of cobalamin or methionine. We investigated this further in liquid cultures of Root65. The strain was preconditioned on defined media with L-methionine supplementation to eliminate carry over cobalamin from LB broth used to routinely culture this organism. Growth curve cultures were established in a 96-well plate with a range of cobalamin concentrations added (0 to 100 nM) as well as a condition with no cobalamin and 250 µM L-methionine. Cultures were grown for 36 hours at 30˚C with OD500 measurements every hour after a brief period of shaking. OD500 measurements at 36 hours of growth (excluding the L-methionine control) were used to fit a four-parameter logistic regression model (LL.4) (R package drc v3.0.1). The model estimated the slope, lower asymptote, upper asymptote, and the half-maximal effective concentration (EC_{50}).

qPCR cycling parameters and sample description

Genomic DNA extracted from *Pseudomonas aeruginosa* – *Pseudoxanthomonas* sp. Root65 co-cultures (Fig. 5) carried out in triplicate were used in technical triplicate qPCR reactions. Each qPCR reaction was carried out in a 10 µL volume with 500 pg of gDNA and 500 nM of a single species primer pair (Table S2). The following cycling program was used: 95°C for 5 minutes followed by 40 cycles of: 95°C for 5 seconds, 60°C for 30 seconds. Technical replicates exhibiting undefined melt curve temperatures (n=2) were removed prior to further analysis. Cq values were converted to DNA concentrations using in-plate

species specific standard curves of gDNA (1 pg/rxn to 1 ng/rxn). Additional control qPCR reactions were conducted to assay for off-target amplification (Fig. S10) and are described below.

Determining qPCR amplification efficiency in mixed gDNA samples

To assess the effect of off-target DNA on qPCR assay efficiency for co-culture quantification, we set up standard curves of known concentrations of both *P. aeruginosa* and Root65 genomic DNA (gDNA) in the presence and absence of off-target DNA. Here, we included 500 pg per reaction of gDNA from the opposite species (500 pg *P. aeruginosa* gDNA for Root65 gDNA curves and vice versa). 500 pg was the maximum DNA concentration used in co-culture qPCR reactions. gDNA concentrations in the standard curve below 0.1 pg were omitted from analysis because they failed to produce Cq values in qPCR assays without off-target gDNA. We determined Cq values at each known gDNA concentration for each primer set and then fit linear regression models with $\log_{10}$-transformed DNA concentration as the predictor and Cq values as the response variable. Models included an interaction term between log DNA concentration and off-target gDNA presence to test whether amplification slopes differed between conditions. Model assumptions were verified using Shapiro-Wilk tests for normality of residuals and Breusch-Pagan tests for homoscedasticity. Because heteroscedasticity was detected (*P. aeruginosa*: chi-squared = 4.00, p = 0.045), a $\log_{10}$ transformation was applied to the response variable to meet model assumptions. Additionally, off-target amplification was tested with a range of genomic DNA concentrations for both species (1 pg – 1 ng) and mismatched primers (i.e. PA14 primers with Root65 genomic DNA and vice versa). The mean Cq for the 1 ng reactions are plotted as dashed lines in Fig S10, and fall above any Cq used for quantification.

Supplemental Tables

Table S1: Strains (*P. aeruginosa*, *E. coli*, and At-RSPHERE)

Table S2: Plasmids & Primers

Table S3: Methionine Synthase Annotations

TS3A – Best hits for methionine synthases with curated IDs and annotation metrics

TS3B – Table of counts for loci with a given annotation per genome

TS3C – Table of locusIDs for hits within each genome
